# Tree‐ring structure determines the temporal coordination between xylem growth and the gain in hydraulic conductivity in the outermost ring

**DOI:** 10.1111/nph.71161

**Published:** 2026-04-24

**Authors:** Laura Fernández‐de‐Uña, Cyrille B. K. Rathgeber, Gonzalo Pérez‐de‐Lis, Anjy N. Andrianantenaina, Matthias Cuntz

**Affiliations:** ^1^ Universidade de Vigo, Department of Plant Biology and Soil Sciences Ourense 32004 Spain; ^2^ AgroParisTech, INRAE, SILVA Université de Lorraine F‐54000 Nancy France; ^3^ iTERRA, BIOAPLIC, Departamento de Botánica Universidade de Santiago de Compostela, Campus Terra 27002 Lugo Spain

**Keywords:** cambial phenology, functional wood anatomy, hydraulic conductivity, intra‐annual dynamics, wood porosity, xylem conduit, xylogenesis

## Abstract

- The study of seasonal xylem hydraulics has predominantly focused on embolism‐induced losses, whereas growth‐driven increases in hydraulic capacity have received little attention.
- We assessed the intra‐annual dynamics of xylem formation and gain of conductivity in the current‐year ring of three species with contrasting tree‐ring structure, sessile oak (*Quercus petraea*, ring‐porous), European beech (*Fagus sylvatica*, diffuse‐porous) and Norway spruce (*Picea abies*, conifer), using micro‐cores collected over 3 yr in north‐eastern France. Ring conductivity loss (indicated by tylose‐occluded vessels) was also examined in both angiosperms.
- Due to the differences in water‐transport efficiency and cavitation vulnerability between earlywood and latewood vessels, oak presented distinct intra‐annual dynamics of current‐ring basal area increment (BAI) and the gain and loss of conductive area (CA), theoretical hydraulic conductivity (*K*_h_) and specific hydraulic conductivity (*K*_s_). Conversely, the intra‐annual gain in BAI, CA and conductivity was largely coordinated in beech and spruce.
- Our results imply that the proportional gain in ring hydraulic capacity could potentially be estimated as a delayed BAI curve in diffuse‐porous and conifer species, but not in ring‐porous species; and the comparison of native conductivity measurements within or across species should account for their timing within the growing season, particularly in ring‐porous species.

## Resumen

- El estudio de los cambios estacionales en la hidráulica del xilema se ha centrado principalmente en las pérdidas causadas por embolismo, mientras que el aumento en la capacidad hidráulica debido al crecimiento ha sido poco estudiado.
- En este trabajo evaluamos las dinámicas intra‐anuales de la formación del xilema y el aumento en la conductividad del anillo del año en curso en tres especies con anatomía de la madera diferenciada: el roble albar (*Quercus petraea*, de anillo poroso), el haya europea (*Fagus sylvatica*, de porosidad difusa) y la pícea común (*Picea abies*, conífera), utilizando micro‐testigos recogidos a lo largo de tres años en el noreste de Francia. También examinamos la pérdida de conductividad de los anillos (indicada por la presencia de vasos ocluidos por tilosis) en ambas angiospermas.
- Debido a las diferencias en eficiencia en el transporte de agua y vulnerabilidad a la cavitación entre los vasos de la madera temprana y los de la madera tardía, el roble presentó dinámicas intra‐anuales diferenciadas en el incremento de área basimétrica (BAI) y el aumento y la disminución de área conductiva (CA), conductividad hidráulica teórica (*K*_h_) y conductividad hidráulica específica (*K*_s_) en el anillo del año en curso. Por el contrario, las ganancias intra‐anuales en BAI, CA y conductividad estaban en gran medida coordinadas en el haya y la pícea.
- Nuestros resultados sugieren que la ganancia de capacidad hidráulica en los anillos de crecimiento podría potencialmente estimarse, de manera proporcional, como una curva de BAI diferida en especies de porosidad difusa y coníferas, pero no en especies de anillo poroso; y que la comparación de mediciones de conductividad nativa dentro de una misma especie o entre especies debe tener en cuenta el momento en el que se realizan dichas observaciones dentro de la estación de crecimiento, especialmente en especies de anillo poroso.

## Résumé

- L’étude saisonnière de l’hydraulique du xylème s’est principalement concentrée sur les pertes dues à l’embolie, tandis que les augmentations de la capacité hydraulique liées à la croissance ont été peu étudiées.
- Nous avons évalué la dynamique intra‐annuelle de la formation du xylème et du gain de conductivité dans le cerne de l’année en cours en trois espèces présentant une anatomie du bois différenciée: le chêne sessile (*Quercus petraea*, cernes à zone poreuse), le hêtre commun (*Fagus sylvatica*, cernes à pores diffus) et l’épicéa commun (*Picea abies*, conifère), en utilisant micro‐carottes prélevées sur trois ans dans le nord‐est de la France. La perte de conductivité des cernes (indiquée par des vaisseaux obstrués par tyloses) a également été examinée chez les deux angiospermes.
- En raison des différences d’efficacité du transport de l’eau et de vulnérabilité à la cavitation entre les vaisseaux de bois initial et de bois final, le chêne présentait des dynamiques intra‐annuelles distinctes concernant l’accroissement de la surface des cernes (BAI) et le gain et la perte de l’aire conductrice (CA), la conductivité hydraulique théorique (*K*_h_) et la conductivité hydraulique spécifique (*K*_s_). Par contre, les gains intra‐annuels de BAI, de CA et de conductivité étaient largement coordonnés chez le hêtre et l’épicéa.
- Nos résultats suggèrent que le gain de la capacité hydraulique des cernes pourrait potentiellement être estimé, de façon proportionnelle, comme une courbe de BAI décalée chez les espèces à pores diffus et les conifères, mais pas chez les espèces à zone poreuse; et la comparaison des mesures de conductivité natives au sein d’une même espèce ou entre espèces devrait tenir compte de leur moment d’observation au cours de la saison de croissance, en particulier chez les espèces à zone poreuse.

## Introduction

Xylem, formed through xylogenesis, has multiple functions, including supporting photosynthetic tissues, ensuring tree stability and transporting water from the roots to the leaves. It consists of a network of interconnected conduits (tracheids and/or vessels), which size, number and distribution, as well as the characteristics of the pits that connect them, regulate its mechanical and hydraulic properties, including water transport efficiency and cavitation resistance (Choat *et al*., 2008; Brodribb *et al*., 2012). In conifers, tracheids provide both water transport and mechanical support, whereas in angiosperms, these functions are primarily performed by vessels and fibers, respectively. Depending on the arrangement of vessels along the ring, angiosperm wood may be broadly classified as ring‐porous, characterized by the significant contrast between wide earlywood vessels and smaller latewood vessels, or diffuse‐porous, with similarly sized vessels. The nonlinear relationship between conduit diameter and conductivity per sapwood area results in a gradient in water transport efficiency, from the more hydraulically limited conduit network of conifers to the highly efficient ring‐porous system (Tyree *et al*., 1991; Sperry *et al*., 2006; Lachenbruch & Mcculloh, 2014). On top of conduit diameter, the use of pits instead of perforation plates to connect conduits makes tracheids further resistant to water flow than vessels (Hacke & Sperry, 2001; Woodruff *et al*., 2010; Choat *et al*., 2012). On the other hand, partly thanks to their large ratio of wall thickness to lumen diameter, tracheids have lower vulnerability to embolism than vessels, although at an increased construction cost (Pittermann *et al*., 2006; Brodribb *et al*., 2012; Hacke *et al*., 2015).

Regardless of the anatomical structure, individual rings within a tree may differ in their hydraulic properties as environmental conditions can affect the timing, rates and duration of xylogenesis processes, and thus the final features of xylem conduits (Cuny *et al*., 2014, 2019; Pérez‐de‐Lis *et al*., 2016b; Buttò *et al*., 2019). Environmental factors can additionally affect the number of cells formed (and thus tree‐ring width) and the proportion of early‐ to late‐wood (Corcuera *et al*., 2004; Cuny *et al*., 2014; Fernández‐de‐Uña *et al*., 2017), in turn influencing the ring's hydraulic properties. The competition for carbon between leaves and wood has also been suggested to affect xylem formation (Cartenì *et al*., 2018), as the early‐to‐latewood transition often coincides with the end of canopy development (Cuny *et al*., 2012; Pérez‐de‐Lis *et al*., 2017; Fernández‐de‐Uña *et al*., 2018). This transition may also be promoted by gibberellins, which induce thicker secondary cell‐walls and are produced by mature leaves (Aloni, 2015). The number of functional rings (i.e. sapwood depth) also determines xylem's hydraulic properties. For example, in ring‐porous species, water transport to the canopy heavily relies on the xylem formed in the current year due to earlywood vessels being generally functional for only one growing season (Cochard & Tyree, 1990; Granier *et al*., 1994; Hacke & Sperry, 2001), whereas in diffuse porous and conifer trees conduits may be functional for many years (Anfodillo *et al*., 1993; Gebauer *et al*., 2008). Nonetheless, sap flow progressively decreases toward the inner sapwood regardless of the wood structure (Anfodillo *et al*., 1993), with most water being transported in the outermost 2 cm (Granier *et al*., 1994; Schäfer *et al*., 2000; Phillips *et al*., 2002; Delzon *et al*., 2004). This highlights the significance of the outermost ring for the tree's water transport. However, the interplay between growth and hydraulic properties, and thus, the implications of ring formation dynamics on xylem water transport, have barely been assessed (but see Jacobsen *et al*., 2015; Copini *et al*., 2019; Valdovinos‐Ayala *et al*., 2022).

Xylogenesis, both in angiosperms and gymnosperms, consists in a sequence of cambial division and cell differentiation processes (cell enlargement and cell wall thickening and lignification) that, in the case of tracheary and vessel elements, finalizes with their programmed death, which renders them functional for water transport. However, conduits progressively lose their function as a result of stress‐induced embolism or the conversion of inner sapwood into heartwood as the tree ages. Irreversibly embolized conduits are then obstructed by gums and tyloses (i.e. outgrowths of adjacent parenchyma cells passing through the pits) (De Micco *et al*., 2016). Therefore, the timing at which tyloses form can provide valuable information on the functional lifespan of xylem conduits (Pérez‐de‐Lis *et al*., 2018).

Species adapt their cambial and leaf phenology to minimize potential damages induced by harmful environmental conditions, such as frosts and droughts, while maximizing functional performance along the growing season. Xylem formation generally starts between March and June in northern temperate forests, often earlier in ring‐porous and conifer species than in coexisting diffuse‐porous species, and finishes by late November, with an earlier cessation in ring‐ and diffuse‐porous species than in conifers (Michelot *et al*., 2012; Martinez del Castillo *et al*., 2016; Fernández‐de‐Uña *et al*., 2018; Marchand *et al*., 2021). Wood porosity also affects the coordination between cambial and leaf phenology, as the onset of xylem enlargement precedes budburst in ring‐porous species but both phenophases generally coincide in diffuse‐porous ones (Michelot *et al*., 2012; Kitin & Funada, 2016; Marchand *et al*., 2021; Savage & Chuine, 2021). Yet, budburst tends to occur before the first vessels mature in both wood types (Kitin & Funada, 2016; Pérez‐de‐Lis *et al*., 2016b; Marchand *et al*., 2021; Valdovinos‐Ayala *et al*., 2022), implying that water transport to the forming canopy is provided by previous‐year xylem. Cambial phenology, and its relationship with leaf phenology, has been increasingly investigated in recent years (Michelot *et al*., 2012; Fernández‐de‐Uña *et al*., 2017, 2018; Pérez‐de‐Lis *et al*., 2017; Marchand *et al*., 2021). Yet, the interplay between xylem formation and conductive function, which is expected to vary significantly with wood porosity, has been seldom studied, mostly focusing on vessel‐bearing species. These pioneering works aimed primarily at the timing at which vessels become functional (Jacobsen *et al*., 2015; Kudo *et al*., 2015; Copini *et al*., 2019; Valdovinos‐Ayala *et al*., 2022) or embolized as indicated by the presence of tyloses (Pérez‐de‐Lis *et al*., 2018). However, the intra‐annual dynamics of stem water transport capacity in the forming ring have never been explored in relation to secondary growth.

Here, we investigated the intra‐annual dynamics in growth and theoretical xylem conductivity of the developing ring, as well as their relationship with leaf phenology, in three species of contrasting tree‐ring structures, *Quercus petraea* (Matt.) Liebl. (ring porous), *Fagus sylvatica* L. (diffuse porous) and *Picea abies* (L.) H. Karst (conifer), growing under the same climatic conditions. We focused on the acquisition of conductive function during xylogenesis and, in the two angiosperm species, their loss of function as indicated by the presence of tyloses. By studying coexisting species, we also aimed at better understanding species‐specific strategies in terms of water transport and use. We hypothesized that the differences across wood types in xylem conductive capacity would result in species‐specific xylem formation strategies, leading to a decoupling between radial growth and hydraulic conductivity gain in ring‐porous species compared with diffuse‐porous and conifer ones. We additionally hypothesized that tree‐ring structure would also determine the coordination between leaf and xylem development.

## Materials and Methods

### Study site

**Fig. 1:**
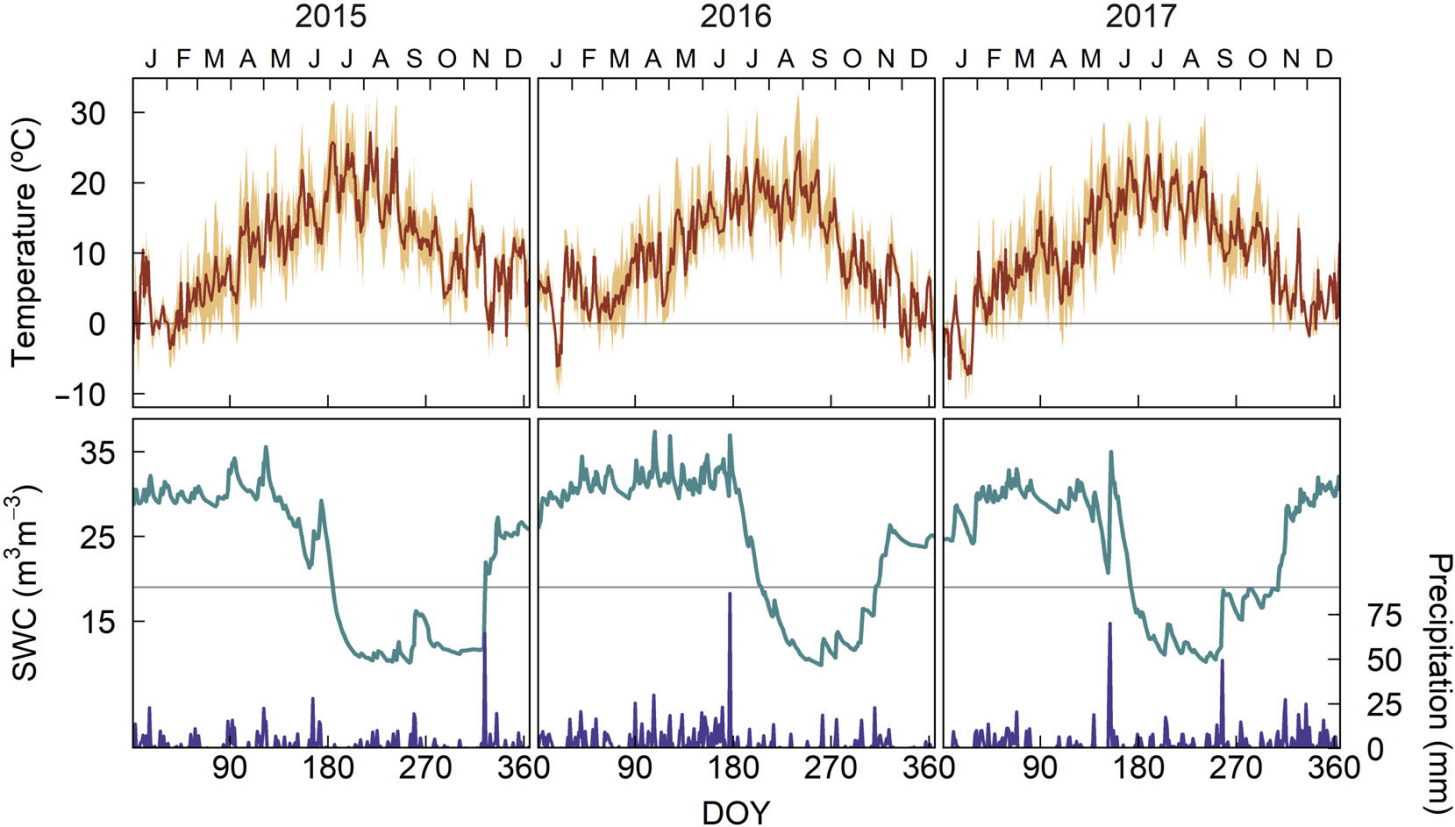
Daily mean temperature, mean soil water content (SWC) within the top 40 cm of soil and precipitation during the study period. Shades in temperature delimit the range between daily minimal and maximal temperatures. The horizontal line in SWC indicates the orientative water deficit threshold (SWC < 0.2 m^3^ m^−3^). DOY, day of the year.

The monitored plots were located at or next to the class 1 ecosystem site FR‐Hes of the Integrated Carbon Observation System (ICOS). The ICOS site was established in 1996 in a 30‐yr‐old European beech (*F. sylvatica*) forest in Hesse, north‐eastern France (48°40′N, 7°05′E, 305 m above sea level; Granier *et al*., 2000, 2008). Other important tree species at the site are sessile oak (*Q. petraea*) and common hornbeam (*Carpinus betulus* L.). In addition, some Norway spruce (*P. abies*) trees have been planted in the vicinity of the stand. The ICOS site has an eddy covariance flux tower that measures ecosystem fluxes (H_2_O, CO_2_ and energy) as well as meteorological and ancillary variables (e.g. air temperature and relative humidity, wind speed and incoming and outgoing radiation) at different heights (up to 35 m) with a half‐hour frequency. In addition, soil temperature and water content are measured at different depths (0–80 cm) at several spots within the site. Mean annual temperature at the site is 10.0°C and mean annual precipitation 889 mm, whereas mean annual temperatures and total precipitation in the study years were, respectively, 10.8°C (warmer than average) and 694 mm (drier than average) in 2015, 10.0°C (average) and 1013 mm (wetter than average) in 2016, and 10.2°C (average) and 928 mm (slightly wetter than average) in 2017 (Fig. 1).

### Wood formation monitoring

Microcores were collected weekly between March and October and bi‐weekly in November and December from seven dominant trees per species at breast height (average tree diameter 25.8 ± 3.2 cm; Supporting Information Table S1). In 2015, beech, oak and spruce were monitored, whereas in 2016 and 2017, wood microcores were only collected for beech and oak as spruce trees were harvested shortly after the first study year. In order to minimize interannual bias due to previous‐year punching injuries, different similarly sized trees were selected each year (Table S1). In addition, microcoring was carried out following an ascending spiral around the trunk, with a separation of several centimeters between samplings (both horizontally and vertically) to limit the effect of previous coring on the wood formation of subsequent samples. Microcores were extracted with a Trephor tool (Rossi *et al*., 2006), conserved in a 50% ethanol solution and stored at 5°C until being processed.

Microcores were dehydrated with increasing concentrations of ethanol and d‐limonene using a tissue processor and embedded in paraffin. Transverse sections of 5 μm were cut with a rotary microtome. To differentiate the xylem cell differentiation stage (i.e. cells undergoing enlargement, wall‐thickening or mature), all microsections were stained with safranine (which binds to lignin) and alcian blue (which binds to other cell wall components) and mounted with Histolaque LMR®. Anatomical sections were photographed with a camera attached to a microscope (AxioImager.M2; Carl Zeiss SAS, Oberkochen, Germany) at ×20 magnification.

### Leaf phenology

Spring leaf phenology (bud swelling, budburst and occasionally different stages of leaf elongation and full leaf maturation) was observed using binoculars on the trees monitored for wood formation on the same dates of microcore collection. Fall phenology (onset of leaf senescence and complete leaf fall) and missing spring leaf maturation data in oak and beech were estimated at the stand level based on daily images from a phenocam on top of the flux tower. For spruce, only bud swelling and budburst were recorded.

### Image analysis and anatomical variable calculation

**Fig. 2:**
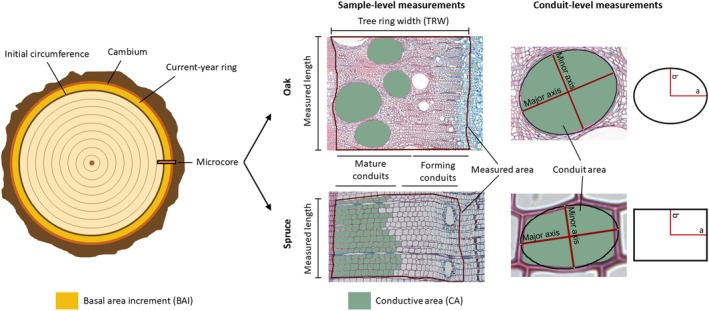
Diagram showing the different variables measured for each sample for oak (upper row) and spruce (lower row). Measurements taken in beech were the same as those in oak.

For each good‐quality section (*c*. 1275, 75%), a measurement area was selected (hereafter, sample). The width of this area was that of the forming ring, from the limit of the previous ring to the vascular cambium. To ensure sufficient conduit size representativity, the length of the measured area was, whenever possible, *c*. 500 μm in beech and spruce and 1000 μm in oak (Fig. 2). Tracheid (spruce) and vessel (beech and oak) lumen areas of all mature conduits contained within each sample were measured, as well as tree ring width (TRW), using Imagej (Schneider *et al*., 2012). The tool ‘Fit ellipse’, which fits the closest ellipse to a conduit shape of the same area, was used to obtain each conduit's major and minor diameters (axes; Fig. 2). Vasicentric tracheid areas were measured in two fully mature oak samples (one from October 2015 and another from October 2016) to assess their contribution to total conductivity; however, due to tracheids having a very small effect on oak's ring conductivity, even compared to latewood vessels (Table S2), they were not measured in the rest of the sample set. This was only tested in oak as beech does not have vasicentric tracheids. Tracheids were considered mature and, therefore, potentially hydraulically active, when cell walls were fully lignified (i.e. no blue coloration was observed in the inner wall), and no cellular content was observed. Vessels were considered mature when cell walls were fully lignified and intervascular pit chambers were completely developed (as these are the last part of the wall to be formed; Kitin & Funada, 2016; Figs S1, S2). In the two angiosperm species, vessels with tyloses at any stage of development were identified to estimate the loss of conductivity due to vessel dysfunction (Fig. S3).

The sample's conductive area (CA) was calculated as the sum of all mature conduit lumen areas within the measured area (Fig. 2). The theoretical xylem hydraulic conductivity of individual xylem conduits (*K*_h_conduit_, kg m s^−1^ MPa^−1^) was calculated assuming an elliptical shape for vessels and latewood tracheids (Eqn 1) and a rectangular shape for earlywood tracheids (Eqn 2) (White, 1991):

(Eqn 1) $$K_{h\_\text{conduit}\_\text{ellipse}}=\frac{\pi \rho {\left(\mathrm{ab}\right)}^{3}}{4\eta \left(a^{2}+b^{2}\right)}\rho$$

where *a* is the major radius (Fig. 2), *b* the minor radius, *η* water viscosity (1.002 × 10^−9^ MPa s at 20°C), and *ρ* is water density (998.2 kg m^−3^ at 20°C);

(Eqn 2) $$K_{h\_\text{conduit}\_\text{rectangle}}=\frac{4ba^{3}}{3\eta }\left(1-\frac{192a}{\pi^{5}b}\sum_{i=1,3,5\ldots }^{\infty }\frac{\tanh\;\left(\mathrm{i\pi b}/2a\right)}{i^{5}}\right)\rho$$

where *a* is half the larger side of the rectangle and *b* half the shorter side (Fig. 2). The measured major and minor diameters of the fitted ellipses (Fig. 2) were used to calculate *a* and *b* in Eqns 1 and 2. For Eqn 1, *a* and *b* were calculated as Major axis/2 and Minor axis/2, respectively (Fig. 2). For Eqn 2, the ratio between the major and minor axes of the ellipse was used as a proxy of the ratio between sides *a* and *b* of the rectangle, which were then calculated as a function of conduit area assuming a rectangular shape: $a=0.5\sqrt{\text{Conduit area}\cdot \left(\text{Major axis}/\text{Minor axis}\right)}$ and $b=0.5\sqrt{\text{Conduit area}\cdot \left(\text{Minor axis}/\text{Major axis}\right)}$. Sample *K*_h_ (*K*_h_sample_) was calculated as the sum of all *K*_h_conduit_ within the measured area, while the specific hydraulic conductivity (*K*_s_, kg m^−1^ s^−1^ MPa^−1^) was calculated as *K*_h_sample_ divided by measured area. To avoid bias due to differing measuring lengths among samples, the initial circumference of the tree (circumference at breast height measured in March minus the estimated bark depth; Fig. 2), was used to calculate basal area increment (BAI), CA and *K*_h_ per ring.

### Data processing and analysis

In order to assess the intra‐annual dynamics in tree‐ring BAI, CA, *K*_h_ and *K*_s_, we fitted for each variable and tree an increasing monotonic Shape Constrained Additive Model (SCAM) with a Gamma distribution as a function of the day of the year (DOY). Given that *K*_s_ in oak may decrease at the end of the growing season due to smaller conduits being formed, generalized additive models (GAM) with a Gamma distribution were fit for this variable in this species instead. Before fitting the models, BAI values after xylem enlargement had ceased were set to the median of the values measured after growth cessation to avoid false fluctuations due to intratree variability around the trunk. Similarly, values of CA, *K*_h_ and *K*_s_ once maturation was completed were set to their respective medians. To assess the loss of vessel conductivity in oak and beech, as indicated by the occurrence of tyloses, we calculated decreasing monotonic SCAMs with a binomial distribution of the ratio between functional (i.e. vessels without tyloses) and total CA, *K*_h_ and *K*_s_ as a function of DOY. Fitted SCAMs and GAMs were used to obtain continuous predictive curves of the intra‐annual dynamics of each trait (BAI, total CA, *K*_h_ and *K*_s_ and functional : total CA, *K*_h_ and *K*_s_). We multiplied each functional : total ratio curve by their corresponding total CA, *K*_h_ and *K*_s_ curves to estimate the intra‐annual dynamics of functional CA, *K*_h_ and *K*_s_. Tree‐level curves were averaged to obtain species‐specific curves per variable and year. To better compare the intra‐annual dynamics among variables and species, we transformed each species‐specific curve to the percentage of its maximum value. The rates at which current‐ring BAI, CA and *K*_h_ increased over the season were calculated as the derivative of their predicted curves. Finally, to further assess the differences in xylem growth and the gain in hydraulic function among species, we calculated the lag in days between a given % BAI and the same % gain in *K*_h_.

The significance of the differences across species and years in wood and leaf phenological dates and final xylem anatomical features (i.e. the tree average once xylogenesis was complete) was tested using generalized linear models (Gamma distribution with an inverse link function) and Tukey *post hoc* tests. Generalized linear mixed models (GLMMs) with Tree as a random effect in the intercept and a Gamma distribution with a log link function were used to test differences in size between vessels with and without tyloses. Linear mixed models (LMMs) with Tree as a random factor on the intercept and the slope were fitted to assess the relationship between % BAI and % *K*_h_. A nonlinear mixed model (NLMM) was also fitted to assess this relationship in oak. We calculated the variance explained by the fixed effects (marginal *R* ^2^) following Nakagawa & Schielzeth (2013), whereas the *R* ^2^ of the fixed effects of the NLMM was estimated as 1 − (sum of squares of residuals/total sum of squares). All analyses were performed in R v.4.3.1 (R Core Team, 2023), using packages scam (Pya, 2023), mgcv (Wood, 2017), lme4 (Bates *et al*., 2015) and nlme (Pinheiro *et al*., 2023) to fit SCAMs, GAMs, GLMMs/LMMs and NLMMs, respectively, and multcomp (Hothorn *et al*., 2008) to calculate *post hoc* multiple comparisons.

## Results

### Intra‐ and inter‐specific differences in wood and leaf phenology

**Fig. 3:**
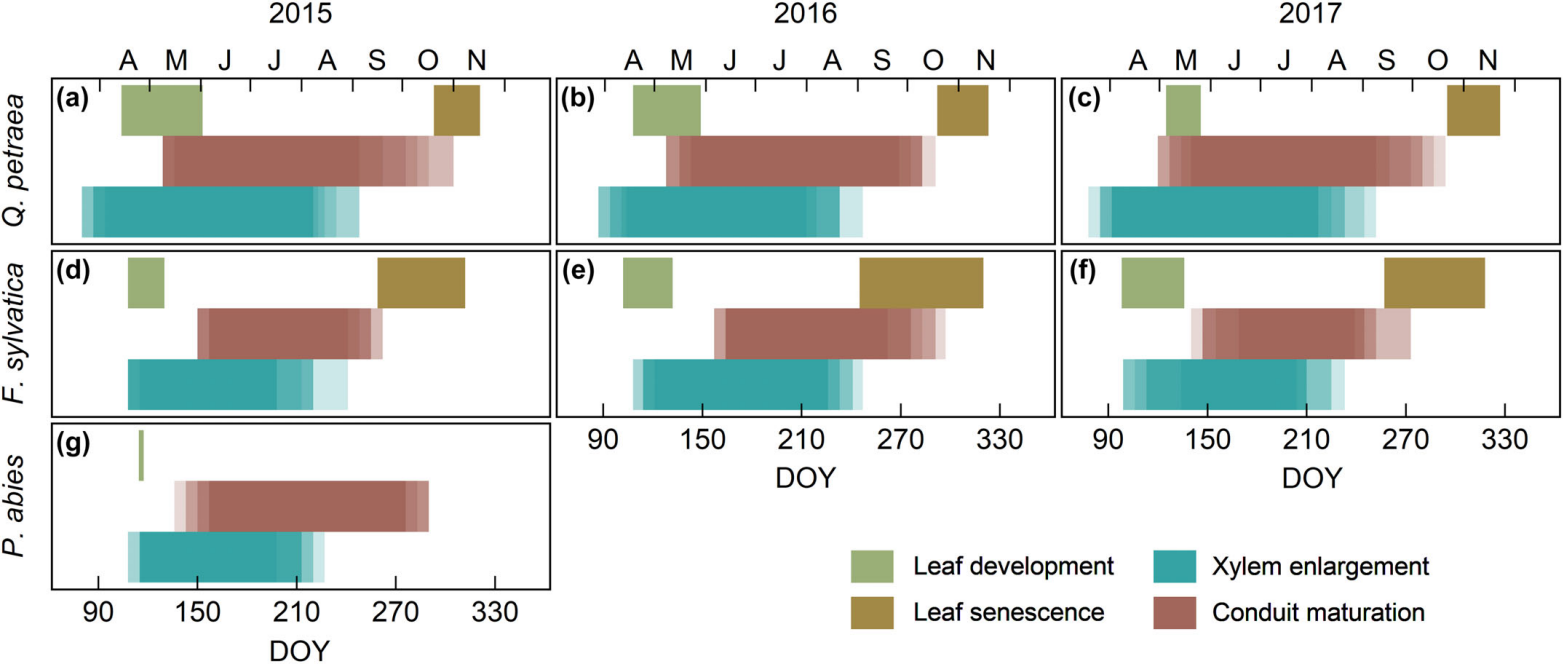
Phenology of the main xylem and leaf developmental processes for each species and study year. (a–c) *Quercus petraea*, (d–f) *Fagus sylvatica*, (g) *Picea abies*. Leaf development marks the period between budburst and full leaf maturation. Leaf senescence marks the period between the onset of leaf yellowing and complete leaf fall. Xylem enlargement marks the period between the observation of the first enlarging cells until the last cell entered wall thickening and lignification. Conduit maturation marks the period since the first mature conduits (tracheids in spruce and vessels in oak and beech) appeared until the last conduits were mature. Color intensity indicates the proportion of trees undergoing that phase. For spruce, only budburst was recorded and this event is thus marked with a vertical line. DOY, day of the year.

Xylogenesis started significantly earlier in oak (late‐March to early‐April) than in beech and spruce (mid‐to‐late‐April of 2015–2017 in beech and 2015 in spruce; Fig. 3). Current‐ring conduits also matured, and thus started to be potentially hydraulically active, earlier in oak (early‐to‐mid May) than in the other two species (late‐May to mid‐June; Fig. 3). The first spruce tracheids took, on average, 5 wk to become mature while it took the first vessels between 4 and 8 wk to become potentially conductive in oak and beech. Budburst occurred in mid‐April in beech and late‐April in spruce, thus concurrently with the start of xylem formation (although depending on the onset of enlargement, budburst could occasionally occur up to 5 wk earlier in beech) and before the new conduits were potentially active (3–6 wk in spruce and 6–10 wk in beech; Fig. 3d–g). In oak, budburst occurred in mid‐April (2015 and 2016) and early‐May (2017), thus 1–3 wk after the onset of enlargement and 3–5 wk before the first current‐year vessels became potentially active in 2015 and 2016 (Fig. 3a,b). In 2017, due to the delay in leaf outing, budburst occurred 5–6 wk after the onset of stem xylem enlargement and between 2 wk before and 1 wk after the maturation of the first vessels (Fig. 3c). In beech, leaf elongation was completed in mid‐May, before the first vessels matured, whereas in oak, leaves reached full expansion in late‐May to early‐June, after the maturation of first‐row vessels. Latewood formation in oak started between mid‐May and mid‐June, around the same time as the completion of leaf expansion (average lag of 2 ± 9 d).

Xylem enlargement ceased significantly earlier in spruce (2015) and beech in 2015 and 2017 (early‐August) than in oak (mid‐to‐late‐August, Fig. 3). Conduit maturation concluded significantly earlier in beech (mid‐September in 2015 and 2017 and early‐October in 2016) than in oak and spruce (mid‐October; Fig. 3). The lignification of vessels was complete on average 2–3 wk earlier than in surrounding tracheids and fibers (data not shown). Leaf senescence started significantly earlier in beech (early–to‐mid‐September) than in oak (mid‐October), concurrently or shortly after the completion of vessel maturation (except in beech in 2016), while complete leaf fall was observed in both species in mid‐November. The duration of ring formation was significantly longer in oak (on average, 143 ± 12 d for ring enlargement and 212 ± 15 d for full ring formation) than in beech (112 ± 16 d and 165 ± 19 d, respectively, although significantly longer in 2016 than in the other 2 yr) and spruce (102 ± 11 d and 172 ± 9 d, respectively).

### Intra‐ and inter‐specific differences in annual growth and hydraulic properties

**Fig. 4:**
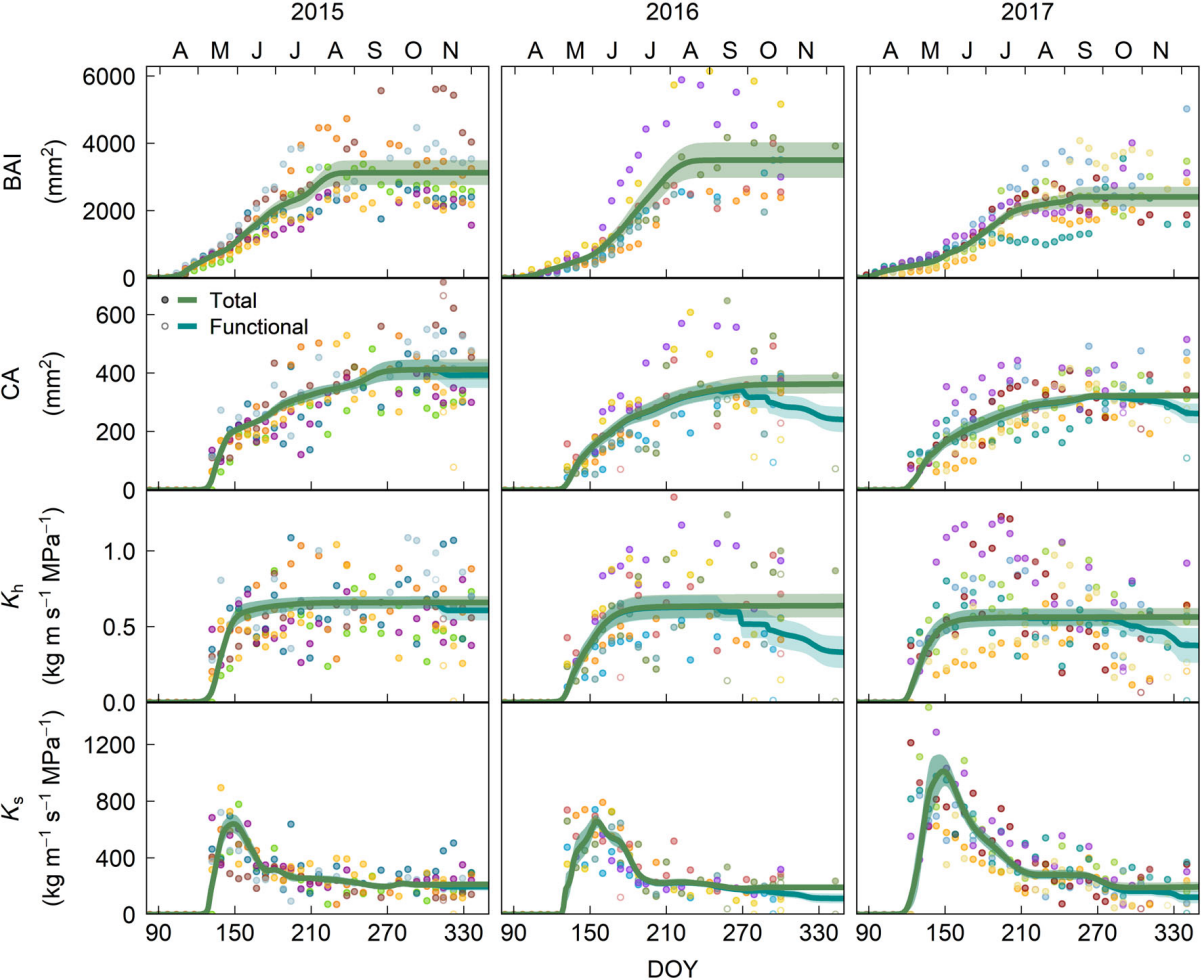
Intra‐annual dynamics in growth (basal area increment – BAI) and total (i.e. vessels with and without tyloses, closed circles) and functional (i.e. vessels without tyloses, open circles) conductive area (CA), theoretical xylem conductivity (*K*_h_) and specific hydraulic conductivity (*K*_s_) for 2015–2017 in *Quercus petraea* in the current‐year ring. Points represent individual trees, with one color per tree. Lines correspond to the average of the tree‐level Shape Constrained Additive Models (BAI, CA and *K*_h_) and generalized additive models (*K*_s_) and shades represent the SE of the mean. DOY, day of the year.

**Fig. 5:**
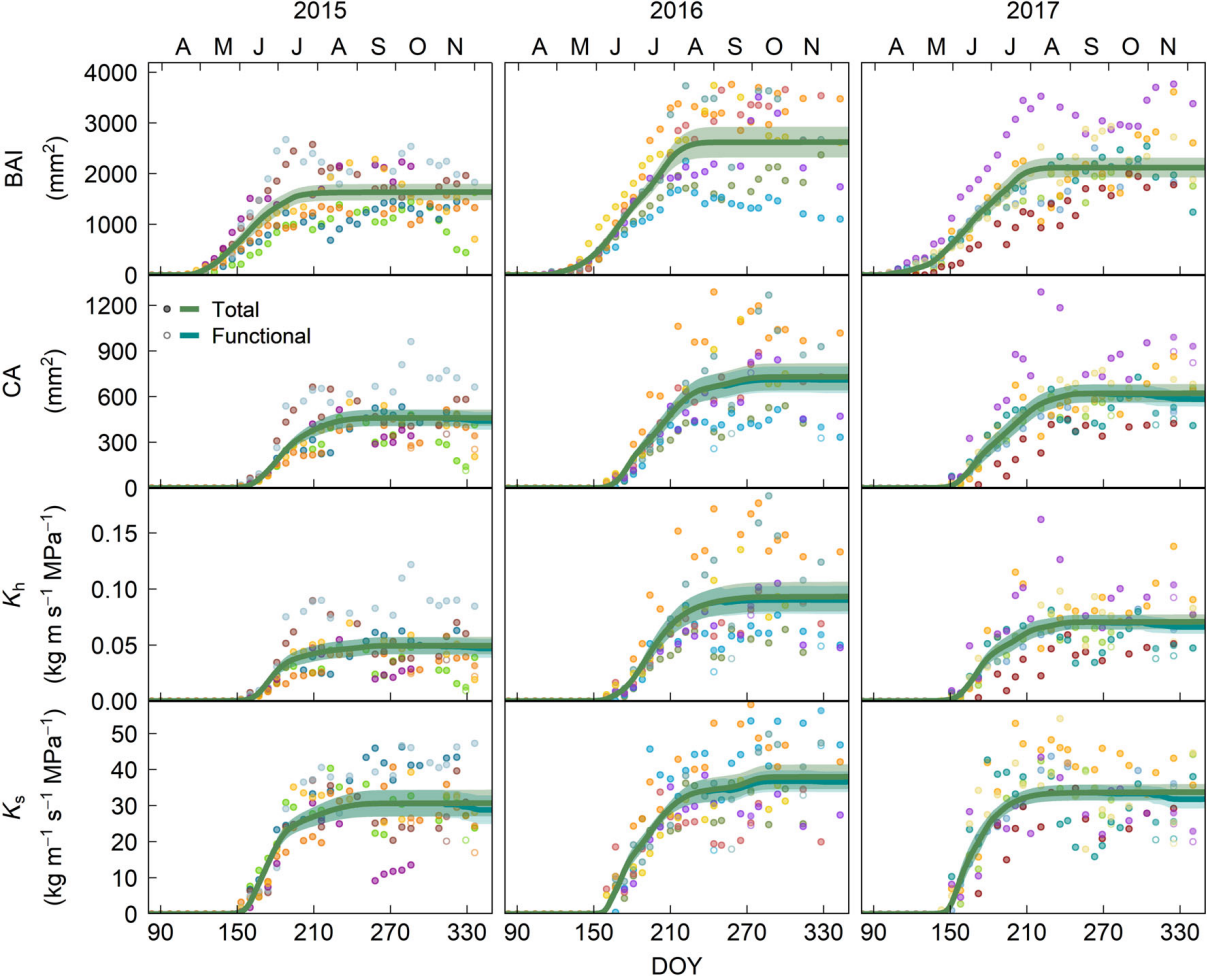
Intra‐annual dynamics in growth (basal area increment – BAI) and total (i.e. vessels with and without tyloses, closed circles) and functional (i.e. vessels without tyloses, open circles) conductive area (CA), theoretical xylem conductivity (*K*_h_) and specific hydraulic conductivity (*K*_s_) for 2015–2017 in *Fagus sylvatica* in the current‐year ring. Points represent individual trees, with one color per tree. Lines correspond to the average of the tree‐level Shape Constrained Additive Models, and shades represent the SE of the mean. DOY, day of the year.

**Fig. 6:**
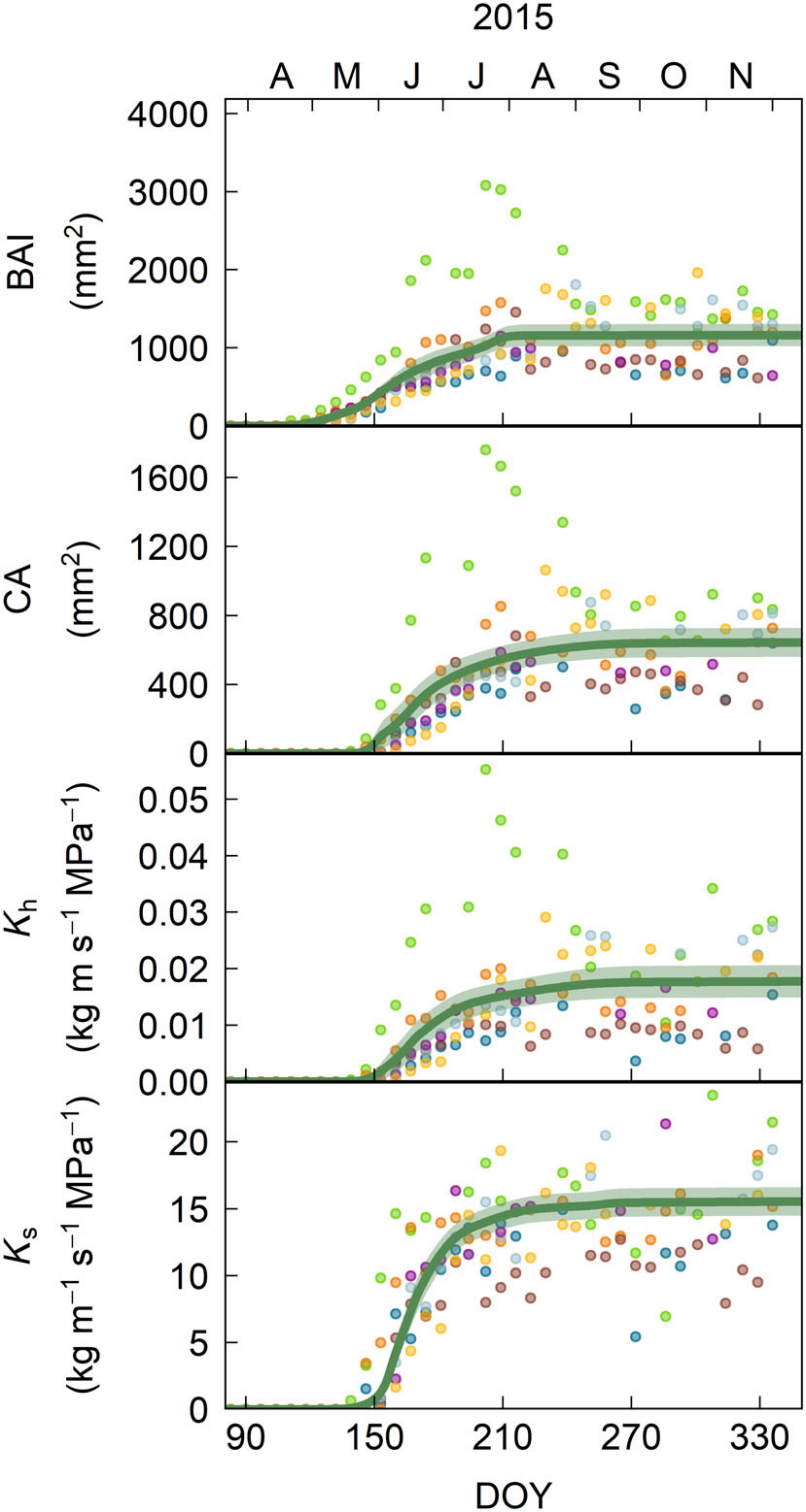
Intra‐annual dynamics in growth (basal area increment – BAI), conductive area (CA), theoretical xylem conductivity (*K*_h_) and specific hydraulic conductivity (*K*_s_) for 2015 in *Picea abies* in the current‐year ring. Points represent individual trees, with one color per tree. Lines correspond to the average of the tree‐level Shape Constrained Additive Models, and shades represent the SE of the mean. DOY, day of the year.

Oak achieved a greater annual BAI (Fig. 4) than beech (Fig. 5) and spruce (Fig. 6), although this difference was only significant in 2015, when all three species were monitored (Fig. S4). This greater BAI was not translated into a higher current‐year ring CA, which was largest in spruce in 2015 and in beech in 2016 and 2017, partly due to the greater proportion of the ring that lumens represent in spruce (55%), compared to beech (29%) and oak (13%; Fig. S4), even accounting for tracheids in the latter (Table S2). Nevertheless, because of the nonlinear relationship between conduit diameter and conductivity (Eqns 1, 2), spruce had a significantly lower theoretical xylem hydraulic conductivity (*K*_h_) and sapwood‐specific hydraulic conductivity (*K*_s_) in the current‐year ring than the other two species. Similarly, oak tracheid lumens represented about one third of oak's CA but < 1% of K_s_ (Table S2), with large earlywood vessels conferring this species the highest ring *K*_h_ and *K*_s_. Interannual anatomical differences were rare in oak, whereas beech trees had greater ring BAI, CA and *K*_h_ in 2016 and 2017 than in 2015, the drier year (Fig. S4).

### Intra‐ and inter‐specific differences in the intra‐annual dynamics of BAI, CA, *K*_h_ and *K*_s_

**Fig. 7:**
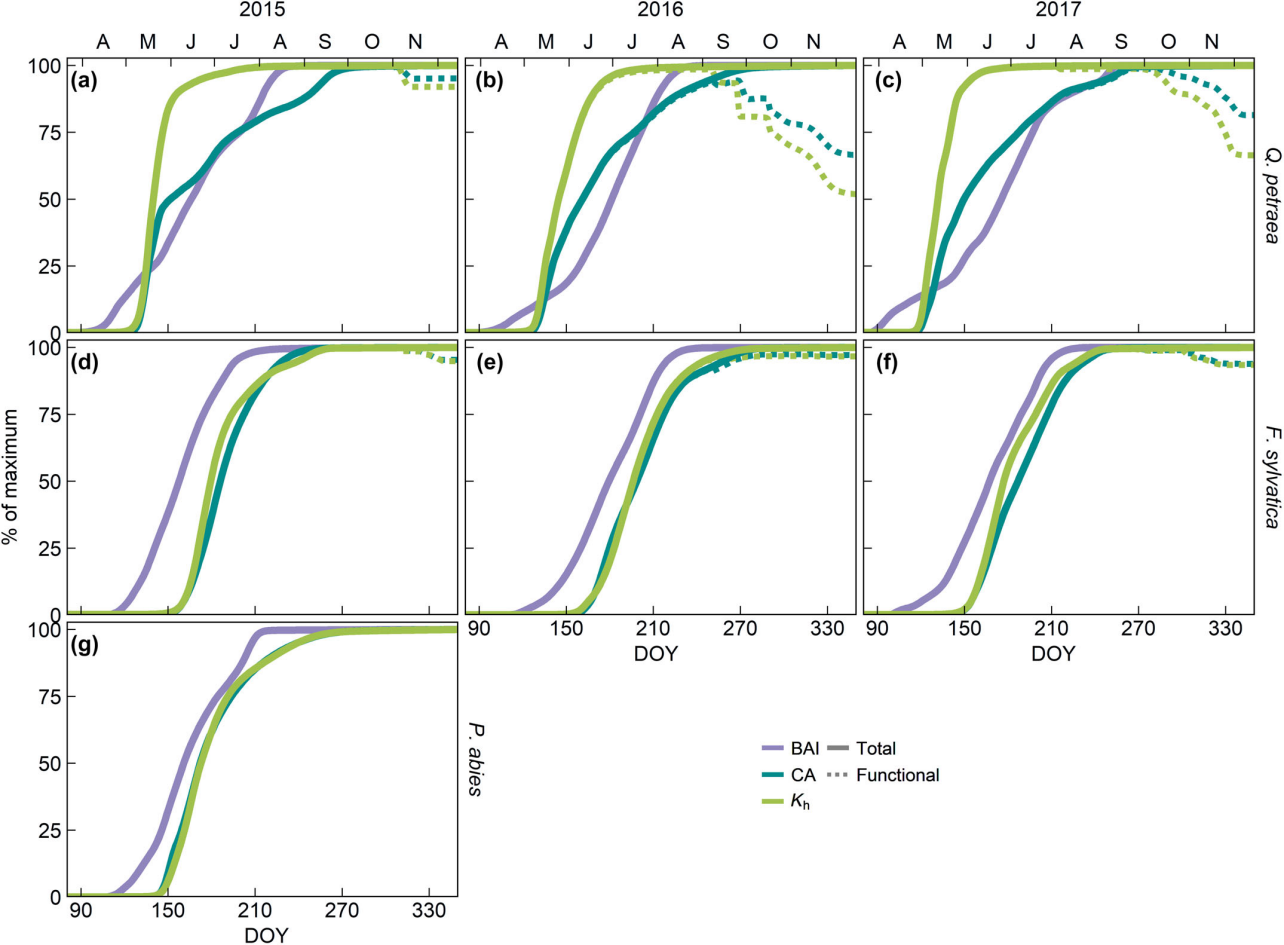
Intra‐annual dynamics in growth (basal area increment – BAI) and total and functional conductive area (CA) and theoretical xylem conductivity (*K*_h_) along the 2015–2017 growing seasons per species (as the % of the maximum value). (a–c) *Quercus petraea*, (d–f) *Fagus sylvatica*, (g) *Picea abies*. DOY, day of the year.

In the three species, all four studied variables (current‐ring BAI, CA, *K*_h_ and *K*_s_) increased along the growing season until reaching a plateau, although this increase was slower in BAI than in the hydraulic traits assessed (Figs 4, 5, 6, 7). The only exception was oak's current‐ring *K*_s_, which increased rapidly early in the season with the formation of large earlywood vessels, reaching its maximum in late‐May to early‐June, but decreased as the growing season progressed (Fig. 4). This was a result of the increase in sapwood area without the addition of new mature vessels as latewood started to develop, followed by the formation of smaller latewood vessels (Fig. S5), gradually reducing the average conductivity on a per area basis (Fig. 4). The difference between earlywood and latewood vessel size also caused a decoupling between the intra‐annual dynamics of oak's BAI, CA and *K*_h_ (Fig. 7a–c). The gain in CA and *K*_h_ peaked in early‐ to mid‐May with the maturation of the first row of earlywood vessels in oak, albeit it was faster in *K*_h_ than in CA (Figs 4, 7a–c, S6). While the gain in basal area was more gradual, growth accelerated in mid‐May when that first row of vessels matured (Figs 7a–c, S6). Once earlywood was completely developed, 55 ± 13% of the ring's final CA and 91 ± 8% of *K*_h_ had been achieved, while only 37 ± 13% of the ring (BAI) had been formed (Figs 4, 7a–c). Afterward, the gain in conductivity remained low until complete ring formation, despite the ongoing basal and CA increase. This resulted in the gain in *K*_h_ preceding that in basal area for most of the ring (average lag of −28 ± 28 d; Fig. S7) and a nonlinear relationship between these two variables (Fig. S8).

On the other hand, the intra‐annual dynamics of current‐year ring CA and *K*_h_ of beech and, particularly, spruce matched within each species for most of the growing season and were always delayed from BAI dynamics (Figs 5, 6, 7). In beech, growth rates started to peak in mid‐to‐late May and remained high for longer in 2016 and 2017 (up to mid‐July) than in 2015 (mid‐June), the drier year (Figs 5, 7d–f, S6). The gains in CA and *K*_h_ were maximal in mid‐to‐late‐June (*K*_h_ in 2016 in mid‐July). The lag between the gains in BAI and *K*_h_ was 21 ± 14 d on average, being shortest in the mid‐part of the ring (Fig. S7). Nonetheless, the relationship between % BAI and % Kh was well represented by a linear relationship (Fig. S8).

In spruce, growth rates peaked at the end of May‐beginning of June, while the gain in CA and *K*_h_ peaked in mid‐June (Figs 6, 7g, S6). The gains in current‐year ring CA and *K*_h_ slowed down in late‐July as earlywood formation was completed and smaller latewood tracheids were formed (Figs. 7g, S5, S6). By then, *c*. 85% of CA and *K*_h_ had been achieved, with the remaining 15% taking *c*. 2.5 months (up to mid‐October) to be acquired. While the limited contribution of latewood to ring conductivity could be expected to cause a slight drop in *K*_s_ gain, this potential reduction was likely buffered by the continued addition of mature tracheids over a constant sapwood area once enlargement ceased. As in beech, the lag between the gains in basal area and *K*_h_ was shortest (6 d) in the middle part of the ring, with an average of 15 ± 10 d (Fig. S7). The relationship between the proportional gains in BAI and *K*_h_ was also linear (marginal *R* ^2^ = 0.923; Fig. S8).

Due to its different wood phenology and anatomy, oak showed distinct intra‐annual CA, *K*_h_ and, to a lesser extent, BAI dynamics compared to beech (2015–2017) and spruce (2015; Figs 7, S9). Growth in 2016 was an exception, when oak's and beech's BAI dynamics were similar after June; oak presented otherwise a gentler incline than the other two species due to its longer period of xylem enlargement (Fig. 3). While beech and spruce had similar spring wood phenology in the studied year (2015), beech BAI gain preceded that of spruce, particularly after mid‐June when spruce's growth rates slowed down. On the other hand, the proportional gains in CA and *K*_h_ occurred earlier in spruce than in beech (Figs 7, S9). Oak's *K*_h_ gain largely preceded that of the other two species, whereas the advancement in CA gain was less pronounced, with the proportional gain of CA in oak dropping below that of spruce in early‐July 2015 and beech in mid‐July 2015 and early‐August 2017 (Figs 7, S9).

### Intra‐annual dynamics in loss of conductivity in oak and beech

Tylose‐occluded vessels appeared in oak in three trees (43% of total) in 2015, four trees (57%) in 2016 and five trees (71%) in 2017, only occurring simultaneously in two or more trees after mid‐October (2017) to early‐November (2015; Fig. S10). This led to a notable loss of functional CA, *K*_h_ and *K*_s_ in the current‐year ring at the end of the growing season in 2016 and 2017, but not in 2015 (Table S3; Figs 4, 7a–c, S11). In some trees, tyloses began forming in earlywood vessels before latewood vessels fully matured; therefore, the current‐year ring never reached its maximal potential conductivity (Figs 4, 7a–c). The presence of tyloses was only slightly lower in beech than in oak (two trees in 2015 – 29% – and four trees in 2016 and 2017 – 57%), generally starting to appear in early‐ to mid‐October (Fig. S10). The percent loss of current‐ring CA, *K*_h_ and *K*_s_ was negligible in beech, with no significant differences across years (Table S3; Figs 5, 7d–f). The loss in these variables was therefore significantly larger in oak than in beech in 2016 and 2017 (Table S3). In both species, tylose‐occluded vessels tended to be larger than nonoccluded ones (Fig. S12), this difference being always significant in beech but only in 2017 in oak.

## Discussion

### Inter‐specific differences in leaf and wood phenology

In all three species (2015 in spruce and 2015–2017 in oak and beech), except oak in 2017, budburst occurred before the first current‐year conduits were potentially hydraulically active, concurring with previous studies (Kitin & Funada, 2016; Pérez‐de‐Lis *et al*., 2016b; Marchand *et al*., 2021; Valdovinos‐Ayala *et al*., 2022). Leaf development and early canopy processes thus relied on previous year xylem for water transport in all three functional types, although this period was shorter in oak than in spruce and beech. This earlier stem vessel formation suggests a more rapid reconnection of the developing leaves to the current‐year vascular system in oak (Savage & Chuine, 2021). The first vessels were mature before full leaf expansion in oak but not in beech (Fig. 3), concurring with previous studies that found fully developed vessel perforations and walls before leaf maturation in ring‐porous species (Kitin & Funada, 2016). The promptness of oak's vessel formation compared to the other two species may be explained by the thinner sapwood depth in this species (5–12 rings) compared to beech (> 30 rings) and spruce (*c*. 55% of basal area; Granier, 1987), as well as the more limited conductive capacity of oak's latewood compared to diffuse‐porous rings. This makes ring‐porous species heavily reliant on current‐year earlywood vessels for efficient water transport to the canopy (Cochard & Tyree, 1990; Granier *et al*., 1994; Hacke & Sperry, 2001).

Xylem enlargement ceased earlier in beech (2015 and 2017) and spruce (2015) than in oak. Growth cessation in beech and spruce coincided with the onset of soil moisture deficits (SWC < 20 m^3^ m^−3^, Fig. 1). The effect of water deficits on beech phenology was supported by the fact that enlargement ceased significantly later in 2016, the wettest year, than in the other two study years (Fig. 3). Beech's greater susceptibility to soil water deficits was also reflected in lower BAI and xylem conductivity in 2015, the drier year (Fig. S4). Conversely, the later cessation in xylem enlargement and lower interannual variability in xylogenesis cessation and ring hydraulic traits may support previous evidence of a higher tolerance to water deficits in oak (Vitasse *et al*., 2019). Of the three species, spruce has the lowest resistance to drought, being negatively affected by summer temperatures and water deficits, particularly at low elevations (Vitasse *et al*., 2019; Jiang *et al*., 2024). While we could not assess interannual variability in spruce, this species only occurs naturally at higher elevations; its lower tolerance to water deficits and high temperatures would thus explain the earlier cessation of tracheid enlargement at our site compared to the nearby Vosges mountains (Cuny *et al*., 2012). Three main, nonmutually exclusive mechanisms have been proposed to halt xylem enlargement under water stress: (1) increasing concentrations of abscisic acid, which causes stomatal closure and inhibits cambial division (Sorce *et al*., 2013); (2) reduction in the turgor levels required to sustain cell growth (Cabon *et al*., 2020); and (3) prioritization of carbon allocation to sustain canopy processes and reserve accumulation over growth (Huang *et al*., 2021).

### Coupling between xylem growth and gain in hydraulic function depends on tree‐ring structure

As hypothesized, contrasting wood porosity led to inter‐specific differences in the intra‐annual dynamics of current‐ring CA and, especially, theoretical xylem hydraulic conductivity (*K*_h_) and specific hydraulic conductivity (*K*_s_), whereas the dynamics of BAI were more affected by cambial phenology (Figs 7, S9). While woody biomass production always lags behind BAI regardless of tree‐ring structure (Cuny *et al*., 2015; Andrianantenaina, 2019), the gain in hydraulic function was delayed or advanced compared to BAI depending on wood porosity (Fig. S7). The significant differences between earlywood and latewood vessel sizes in oak led to an uncoupling between the intra‐annual dynamics of current‐year ring BAI, CA, *K*_h_ and *K*_s_ within this species (Figs 4, 7), which were also different from those of the other two species (Fig. S9). Oak's intra‐annual dynamics in growth and conductivity gain revealed, for a comparable increment in xylem area, a strong investment in hydraulic efficiency early in the growing season, and in hydraulic safety later on, following the seasonal gradient in water availability (Zanne *et al*., 2006; Fig. 1). As a result, oak trees reached up to 98% of total current‐year ring conductivity by early June. Low earlywood density (large proportion of tissue occupied by cell lumina) may additionally minimize carbon allocation to wood formation while the canopy is not yet fully functional and stored carbohydrates must be used for xylem and canopy development (Pérez‐de‐Lis *et al*., 2016a; Cartenì *et al*., 2018; Noyer *et al*., 2023). Once the first earlywood vessels became active, the boosted water transport capacity induced higher growth rates, as observed after the first earlywood vessels matured (Figs 7, S6). Growth rates may have also been enhanced thanks to progressively increasing carbon availability, as developing leaves may photosynthesize well before reaching full expansion (Keel & Schädel, 2010). Latewood, which is much denser than earlywood, began forming when leaves matured and were thus fully functional, allowing greater carbon allocation to xylem formation (Cartenì *et al*., 2018). The more cavitation‐resistant latewood vessels can also be active for several years (Umebayashi *et al*., 2010). Thus, they not only play a significant role during the current growing season in case of earlywood vessel dysfunction but also support budburst and early leaf development in spring as current‐year earlywood vessels are not yet functional (Kudo *et al*., 2018; Copini *et al*., 2019).

On the other hand, BAI was similarly translated into increases in current‐year ring CA, *K*_h_ and *K*_s_ in beech and spruce as a result of their more homogenous conduit distribution along the ring (i.e. lower coefficient of variation; Fig. S5). Latewood contributed little to the ring's conductivity in spruce, due to its lower width and smaller tracheid lumen size. Even though tracheids < 6 μm in diameter, such as some spruce's latewood tracheids, may only be able to conduct water radially (Canny, 1991), conifer latewood cells play a significant role in water storage, as well as in structural support thanks to their thick cell walls (Domec & Gartner, 2002a). Thicker walls explain the longer time spans needed for latewood maturation.

The linear relationship between the proportional gain in BAI and that of *K*_h_ in beech and spruce (Fig. S8) suggests that the intra‐annual gain in current‐year ring *K*_h_ could be potentially estimated in diffuse‐porous and conifer species as a delayed BAI curve using final ring measurements of *K*_h_. However, in ring‐porous species, the nonlinear relationship between the proportional gain in BAI and *K*_h_ as a result of *K*_h_ gain preceding BAI hinders this estimation. Although we were able to accurately model the proportional gain in current‐year ring *K*_h_ as a function of that of BAI in oak (Fig. S8), this relationship cannot be extrapolated as it is largely influenced by the proportion of earlywood and latewood. For instance, in trees with a thin latewood, such as those under drought or high competition (Corcuera *et al*., 2004, 2006; Fernández‐de‐Uña *et al*., 2017), earlywood would represent a larger percentage of BAI and an even larger % of *K*_h_ than in our samples, and thus *K*_h_ would reach the 100% gain at higher % BAI.

### Seasonal conductivity loss in relation to ring porosity

Both beech and oak experienced vessel dysfunction in the current‐year ring. Vulnerability to embolism increases with vessel size (Pittermann & Sperry, 2003; Sperry *et al*., 2006; Hacke *et al*., 2023; Jacobsen & Pratt, 2023). We accordingly found that wider vessels tended to be those occluded by tyloses in both species. Tylose‐occluded vessels were also larger in oak in 2016 and 2017, when trees experienced a greater loss of conductivity (Figs 7, S12). The loss of conductivity in the current‐year ring was greater in oak than in beech, particularly in 2016 and 2017 (Table S3). The safety vs efficiency tradeoff entails that hydraulic conductivity is higher in ring‐porous than in diffuse porous species, but also declines more abruptly under increasingly negative pressures (Cochard & Tyree, 1990; Hacke *et al*., 2006; Taneda & Sperry, 2008). Through the production of large, highly‐efficient but vulnerable vessels early in the season, ring‐porous oaks may thus follow a *sacrificial* strategy that promotes water transport and minimizes carbon investment into wood formation during canopy development at the expense of higher embolism vulnerability later on (Hacke *et al*., 2006, 2017; Jacobsen & Pratt, 2023). Conversely, diffuse‐porous beech prioritized safety over the entire growing season, at the cost of efficiency (Hacke *et al*., 2006; Zanne *et al*., 2006; Jacobsen & Pratt, 2023), through the formation of smaller vessels, which resulted in a negligible loss of conductivity in the current‐year ring (Fig. 7; Table S3). Fibers surrounding vessels have been suggested to reinforce vessel walls and thus increase their resistance to embolism (Sperry *et al*., 2006; Jacobsen *et al*., 2015), which may contribute to the fewer tyloses found in beech and oak latewood vessels compared to oak earlywood vessels, which are mostly surrounded by tracheids. *Fagus sylvatica* shows stronger stomatal control and decreased water use under water stress compared to *Q. petraea* (Aranda *et al*., 2005; Urli *et al*., 2013), which may have further protected beech vessels from drought‐induced cavitation during the summer. Nevertheless, oak's current‐year ring *K*_h_ remained higher than that of beech (Fig. S11). The high *K*_h_ of the last ring may therefore partly compensate the lower sapwood depth of oak compared to beech, consistent with studies that found that ring‐porous species retained *K*_h_ levels as high as those of non‐embolized diffuse‐porous stems even after a > 80% sapwood conductivity loss (Hacke *et al*., 2006).

In both beech and oak, tyloses started to be common in the current ring between mid‐October and mid‐November (Figs 7 and S10), coinciding with the first frosts (Fig. 1). Conversely, Pérez‐de‐Lis *et al*. (2018) found abundant tyloses in *Quercus robur* and *Q. pyrenaica* as early as June under a more Mediterranean climate. Although cavitation fatigue may have accumulated through the summer (Hacke *et al*., 2001; Umebayashi *et al*., 2019), irreversible cavitation in both beech and oak was likely more associated with autumn frosts rather than summer water stress, indicating their vessels are well adapted to withstand the moderately dry summer conditions of our site. Cochard *et al*. (1992) found, at a nearby location, that the conductivity loss in *Q. petraea* twigs was below 20% during summer, even under a water‐stress treatment, but increased to almost 90% after the first hard frost in early November. *Fagus sylvatica* twigs also showed a greater loss of conductivity during winter (up to 80%), which was partly recovered during spring and summer due to vessel refilling and the formation of new vessels (Cochard *et al*., 2001). The lower values of conductivity loss observed here compared to previous studies may be explained by (1) hydraulic vulnerability segmentation, which makes distal tissues such as twigs more vulnerable to cavitation than trunks (Johnson *et al*., 2016); (2) we only monitored irreversible loss (i.e. no possibility of recovery through refilling); and (3) we only assessed the last ring, while older xylem is more vulnerable to cavitation because of the accumulation of hydraulic damage and reduced conduit refilling capacity due to its greater distance from the phloem (Canny, 1997; Domec & Gartner, 2001, 2002b; Fukuda *et al*., 2015). Despite the associated loss of conductivity, particularly in oak, the effect of xylem's cavitation on canopy fluxes and overall tree functioning was likely insignificant in our site as the greater conductivity decline occurred after leaves had started senescence (Figs 3, 7).

### Using anatomical features to assess conduit functionality

Our results imply that the time when conductivity measurements are taken within the growing season can have a significant effect on the values obtained, particularly considering that hydraulic conductivity and percent loss of conductivity measurements are performed in small branches or stems containing very few rings, where the forming ring may thus have a significant weight on total sapwood conductivity. This is especially true in ring‐porous species, where most water transport occurs in the last ring, and which *K*_s_ steeply reaches its maximum early in the season but significantly decreases later on. This biases native conductivity measurements and hinders the comparison of measures taken at different timings or between species with different phenology. Our anatomical approach may thus facilitate a more integrative understanding of the seasonal changes in xylem function in the current‐year ring, particularly in the stems of mature individuals. This information could be complemented by data obtained through techniques such as active‐xylem staining, magnetic resonance imaging or X‐ray micro‐computed tomography (micro‐CT), which allow the observation of vessel functionality *in vivo* (Fukuda *et al*., 2015; Jacobsen *et al*., 2015, 2018; Kudo *et al*., 2015; Copini *et al*., 2019; Valdovinos‐Ayala *et al*., 2022). These approaches are, however, generally limited to small‐diameter samples (either saplings, vines and shrubs or shoots and branches of larger individuals) and imply the destruction of the individual or organ under study, even in large trees (Kudo *et al*., 2015), whereas our anatomical approach allowed us to monitor the hydraulic function of the current‐year ring on the stems of mature individuals.

It has been suggested that conduit maturation may not properly reflect functionality due to the potential delay between when conduits appear mature and when they actually start to contribute to water flow (Jacobsen *et al*., 2018). However, we found that the first oak and beech vessels took 4–8 wk to mature, agreeing with vessel‐staining studies performed in *Quercus serrata* (about a month, Kudo *et al*., 2015), *Q. rubra* (4 wk), *Acer rubrum* or *Populus balsamifera* (7 wk; Valdovinos‐Ayala *et al*., 2022). Valdovinos‐Ayala *et al*. (2022) observed mature vessels 2–6 wk after the onset of enlargement and a delay of up to 4 wk between vessels appearing mature and dye‐identified hydraulic function. Detailed observation of vessel wall lignification, with a particular focus on intervascular pit chambers (Fig. S1), may have allowed for a more accurate identification of complete vessel maturation than previously used methods, thus reducing the potential gap between mature conduit identification and conductive function.

The identification of tyloses can be a useful approach to investigate xylem loss of function in the stem of mature trees (Pérez‐de‐Lis *et al*., 2018), as their presence, regardless of their developmental stage, indicates permanent conduit dysfunction. Yet, this approach has limitations. On the one hand, tyloses must develop to anatomically discern the loss of hydraulic function. This likely hindered the identification of some embolized vessels, particularly recently cavitated ones, and precludes using the method in species that do not or rarely form tyloses after injury, infection or drought‐ or frost‐induced functional loss, such as conifers (De Micco *et al*., 2016). Therefore, from this perspective, we could have underestimated the extent of vessel dysfunction. On the other hand, regular microcoring could have induced tylosis development (De Micco *et al*., 2016). However, weekly microcores were separated by several centimeters, and no reaction wood was observed. Furthermore, microcore extraction should have produced a more regular pattern of vessel occlusion across trees, within and between years. Therefore, although we do not discard the presence of injury‐related tyloses, it is unlikely that mechanical injuries were the main cause behind tylosis formation in this study. Nonetheless, special attention should be paid to the separation between samplings to prevent the formation of punching‐induced tyloses and ensure reliable records of naturally occurring xylem dysfunction in the forming ring. Moreover, our method only allows monitoring the forming ring, despite older xylem being more vulnerable to cavitation (Canny, 1997; Domec & Gartner, 2001, 2002b; Fukuda *et al*., 2015). Finally, actual conductivity may be as low as 20% of the theoretical conductivities assessed here, as calculations do not account for the resistance of the pits and perforation plates connecting tracheids and vessel elements (Tyree & Ewers, 1991). Despite these limitations, frequent monitoring of conduit formation and maturation, as well as their eventual loss of functionality as indicated by the presence of tyloses, provides, as shown here, valuable information on the intra‐annual dynamics of the current‐ring function, which plays a significant role in water transport (Granier *et al*., 1994; Schäfer *et al*., 2000; Phillips *et al*., 2002; Delzon *et al*., 2004).

### Conclusions

This work shows that current‐year ring hydraulic properties are not static but change along the growing season, both as a result of the formation of new conduits and the loss of their functionality as indicated by the presence of tyloses. Monitoring wood formation can therefore be a useful tool to assess not only intra‐annual changes in growth but also in xylem functions such as hydraulic conductivity. Our analysis showed large differences among species in their intra‐annual dynamics of current‐year ring BAI, conductive area, theoretical hydraulic conductivity (*K*_h_) and specific conductivity (*K*_s_). In the three studied species, *Q. petraea*, *F. sylvatica* and *P. abies*, the gain in BAI was generally slower than the gain in CA and *K*_h_. Conduit‐size homogeneity along the ring, rather than vessel vs tracheid structure, leaf habit (i.e. deciduous vs evergreen) or phylogenetic proximity (Fagaceae vs Pinaceae) determined the current‐year ring dynamics of these traits, being more dissimilar in ring‐porous oak compared to diffuse‐porous beech and coniferous spruce. The resulting differences in xylem vulnerability to embolism in beech and oak also affected the dynamics in the loss of CA and hydraulic conductivity. Our results suggest that the proportional gain in current‐year ring *K*_h_ could be potentially estimated as a delayed BAI curve in diffuse‐porous and conifer species. However, this would be more challenging in ring‐porous species because the gain in conductivity precedes that of BAI once the first vessels have matured. Our findings also imply that measurements of xylem conductivity and percent loss of conductivity should be interpreted in the context of growth‐related changes in hydraulic capacity over the growing season. This is particularly true in cases where the forming ring may have a significant weight on total sapwood conductivity, such as in ring‐porous species or small‐diameter samples.

## Competing interests

None declared.

## Author contributions

LF‐U, CBKR and MC conceived and designed the research. ANA performed sample imaging. LF‐U measured the samples, analyzed the data (with assistance of GP‐L) and wrote the manuscript with contributions from all authors.

## Disclaimer

The New Phytologist Foundation remains neutral with regard to jurisdictional claims in maps and in any institutional affiliations.

## Supporting information


**Fig. S1** Criteria used to identify mature vessels in transverse sections, illustrated for *Quercus petraea*.
**Fig. S2** Mature and developing vessels in a transverse (upper) and a radial (lower) section of *Fagus sylvatica* (June 2017).
**Fig. S3** Tyloses in angiosperm samples.
**Fig. S4** Anatomical characteristics of the current‐year ring once the ring was formed in *Quercus petraea*, *Fagus sylvatica* and *Picea abies* for each study year (2015–2017).
**Fig. S5** Changes in mean conduit diameter, vessel composition and coefficient of variation of conduit area (CV) along the 2015 growing season in *Quercus petraea*, *Fagus sylvatica* and *Picea abies*.
**Fig. S6** Daily gain in basal area increment (ΔBAI), conductive area (ΔCA) and theoretical xylem conductivity (Δ*K*_h_) along each study year (2015–2017) in *Quercus petraea*, *Fagus sylvatica* and *Picea abies*.
**Fig. S7** Lag in days between the gain in basal area increment (BAI) and theoretical xylem conductivity (*K*_h_) per species and study year.
**Fig. S8** Relationship between the gain in basal area (BAI) and theoretical xylem conductivity (*K*_h_) per species and study year.
**Fig. S9** Mean changes in basal area increment (BAI), conductive area (CA) and theoretical xylem conductivity (*K*_h_) in absolute values and as a percentage of the species‐specific maximum along each study year in *Quercus petraea*, *Fagus sylvatica* and *Picea abies*.
**Fig. S10** Percentage of *Quercus petraea* (above) and *Fagus sylvatica* (below) trees that presented vessels with tyloses on each sampling date.
**Fig. S11** Total vs functional conductive area (CA), theoretical xylem conductivity (*K*_h_) and specific hydraulic conductivity (*K*_s_) in *Quercus petraea* (above) and *Fagus sylvatica* (below) trees for each study year.
**Fig. S12** Vessel diameter of vessels with and without tyloses in oak's earlywood and beech.
**Table S1** Diameter (mean ± SD, cm) of the sampled trees per species and study year.
**Table S2** Contribution (mean ± SD) of earlywood vessels, latewood vessels and tracheids to total ring area, ring conductive area and ring‐specific hydraulic conductivity (*K*_s_) in *Quercus petraea*.
**Table S3** Reduction (in %) in functional conductive area (CA), theoretical xylem conductivity (*K*_h_) and specific hydraulic conductivity (*K*_s_) per species and study year as a result of vessel dysfunction as indicated by the presence of tyloses.
Please note: Wiley is not responsible for the content or functionality of any Supporting Information supplied by the authors. Any queries (other than missing material) should be directed to the *New Phytologist* Central Office.

## Data Availability

The data that support the findings of this study are openly available in Zenodo at doi: 10.5281/zenodo.19071276.
