# Supplementary material for: Tree‐ring structure determines the temporal coordination between xylem growth and the gain in hydraulic conductivity in the outermost ring
Source: New Phytol. 2026 Apr 24;250(6):3732–46. doi: 10.1111/nph.71161 (PMC13193432; doi:10.1111/nph.71161)
Supplement: Supplementary file 1 — Fig. S1 Criteria used to identify mature vessels in transverse sections, illustrated for Quercus petraea. Fig. S2 Mature and developing vessels in a transverse (upper) and a radial (lower) section of Fagus sylvatica (June 2017). Fig. S3 Tyloses in angiosperm samples. Fig. S4 Anatomical characteristics of the current‐year ring once the ring was formed in Quercus petraea, Fagus sylvatica and Picea abies for each study year (2015–2017). Fig. S5 Changes in mean conduit diameter, vessel composition and coefficient of variation of conduit area (CV) along the 2015 growing season in Quercus petraea, Fagus sylvatica and Picea abies. Fig. S6 Daily gain in basal area increment (ΔBAI), conductive area (ΔCA) and theoretical xylem conductivity (ΔK h) along each study year (2015–2017) in Quercus petraea, Fagus sylvatica and Picea abies. Fig. S7 Lag in days between the gain in basal area increment (BAI) and theoretical xylem conductivity (K h) per species and study year. Fig. S8 Relationship between the gain in basal area (BAI) and theoretical xylem conductivity (K h) per species and study year. Fig. S9 Mean changes in basal area increment (BAI), conductive area (CA) and theoretical xylem conductivity (K h) in absolute values and as a percentage of the species‐specific maximum along each study year in Quercus petraea, Fagus sylvatica and Picea abies. Fig. S10 Percentage of Quercus petraea (above) and Fagus sylvatica (below) trees that presented vessels with tyloses on each sampling date. Fig. S11 Total vs functional conductive area (CA), theoretical xylem conductivity (K h) and specific hydraulic conductivity (K s) in Quercus petraea (above) and Fagus sylvatica (below) trees for each study year. Fig. S12 Vessel diameter of vessels with and without tyloses in oak's earlywood and beech. Table S1 Diameter (mean ± SD, cm) of the sampled trees per species and study year. Table S2 Contribution (mean ± SD) of earlywood vessels, latewood vessels and tracheids to total ring area, ring condu [file NPH-250-3732-s001.pdf]

***New Phytologist* Supporting Information**

Article title: Tree-ring structure determines the temporal coordination between xylem growth and the gain in hydraulic conductivity in the outermost ring

Authors: Laura Fernández-de-Uña, Cyrille B. K. Rathgeber, Gonzalo Pérez-de-Lis, Anjy N. Andrianantenaina, Matthias Cuntz

Article acceptance date: 15 March 2026

The following Supporting Information is available for this article:

Fig. S1 Criteria used to identify mature vessels in transverse sections, illustrated for *Quercus petraea*.

Fig. S2 Mature and developing vessels in a transverse (up) and a radial (bottom) section of *Fagus sylvatica* (June 2017).

Fig. S3 Tyloses in angiosperm samples.

Fig. S4 Anatomical characteristics of the current-year ring once the ring was formed in *Quercus petraea*, *Fagus sylvatica* and *Picea abies* for each study year (2015–2017).

Fig. S5 Changes in mean conduit diameter, vessel composition and coefficient of variation of conduit area (CV) along the 2015 growing season in *Quercus petraea*, *Fagus sylvatica* and *Picea abies*.

Fig. S6 Daily gain in basal area increment (ΔBAI), conductive area (ΔCA) and theoretical xylem conductivity (ΔK_h) along each study year (2015–2017) in *Quercus petraea*, *Fagus sylvatica* and *Picea abies*.

Fig. S7 Lag in days between the gain in basal area increment (BAI) and theoretical xylem conductivity (K_h) per species and study year.

Fig. S8 Relationship between the gain in basal area (BAI) and theoretical xylem conductivity (K_h) per species and study year.

Fig. S9 Mean changes in basal area increment (BAI), conductive area (CA) and theoretical xylem conductivity (K_h) in absolute values and as a percentage of the species-specific maximum along

each study year in *Quercus petraea*, *Fagus sylvatica* and *Picea abies*.

Fig. S10 Percentage of *Quercus petraea* (above) and *Fagus sylvatica* (below) trees that presented vessels with tyloses on each sampling date.

Fig. S11 Total vs functional conductive area (CA), theoretical xylem conductivity (K_h) and specific hydraulic conductivity (K_s) in *Quercus petraea* (above) and *Fagus sylvatica* (below) trees for each study year.

Fig. S12 Vessel diameter of vessels with and without tyloses in (a) oak's earlywood and (b) beech.

Table S1 Diameter (mean ± standard deviation, cm) of the sampled trees per species and study year.

Table S2 Contribution (mean ± standard deviation) of earlywood vessels, latewood vessels and tracheids to total ring area, ring conductive area and ring specific hydraulic conductivity (K_s) in *Quercus petraea*.

Table S3 Reduction (in %) in functional conductive area (CA), theoretical xylem conductivity (K_h) and specific hydraulic conductivity (K_s) per species and study year as a result of vessel dysfunction as indicated by the presence of tyloses.

Fig. S1 Criteria used to identify mature vessels in transverse sections, illustrated for *Quercus petraea*. (a) Lignified walls, including pit chambers (fully stained with safranine); (b) Not fully lignified walls; (c) Vessels considered mature (left) and not yet mature (centre and right) based on the degree of wall lignification. Note that the vessel in the middle has most of the wall lignified but pits are still blue, indicating the wall there is not yet fully lignified.

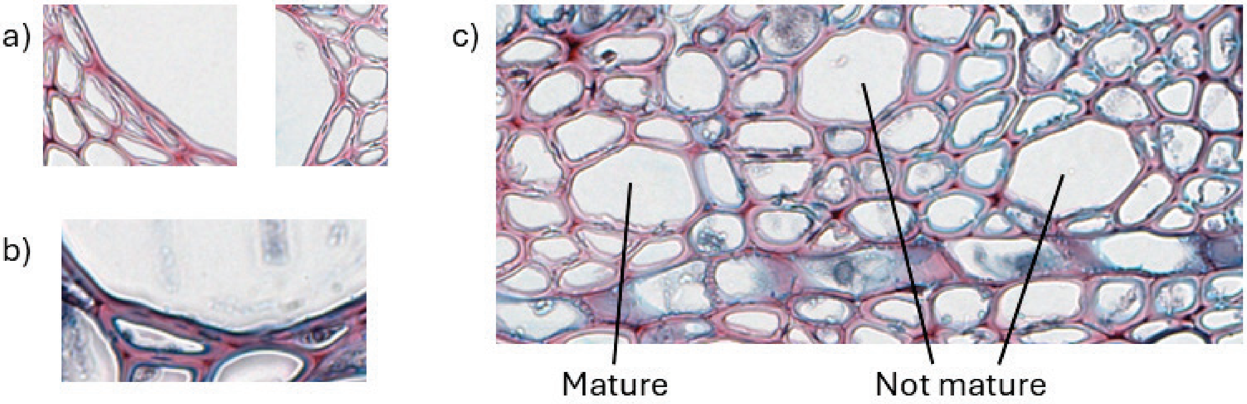

Fig. S2 Mature and developing vessels in a transverse (up) and a radial (bottom) section of *Fagus sylvatica* (June 2017). Vessels considered matured based on their anatomical features as observed on the transversal sections (lignified walls, including pit chambers, and no cell content) presented fully formed perforation plates.

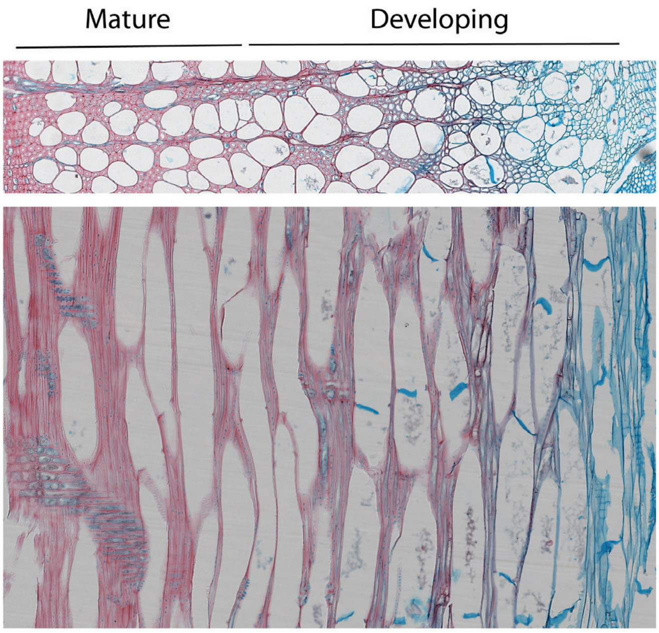

Fig. S3 Tyloses in angiosperm samples. (a) and (b) Tyloses in *Quercus petraea* in early stages of development; (c) Fully formed tyloses in *Q. petraea*; (d) Fully formed tyloses in *Fagus sylvatica*.

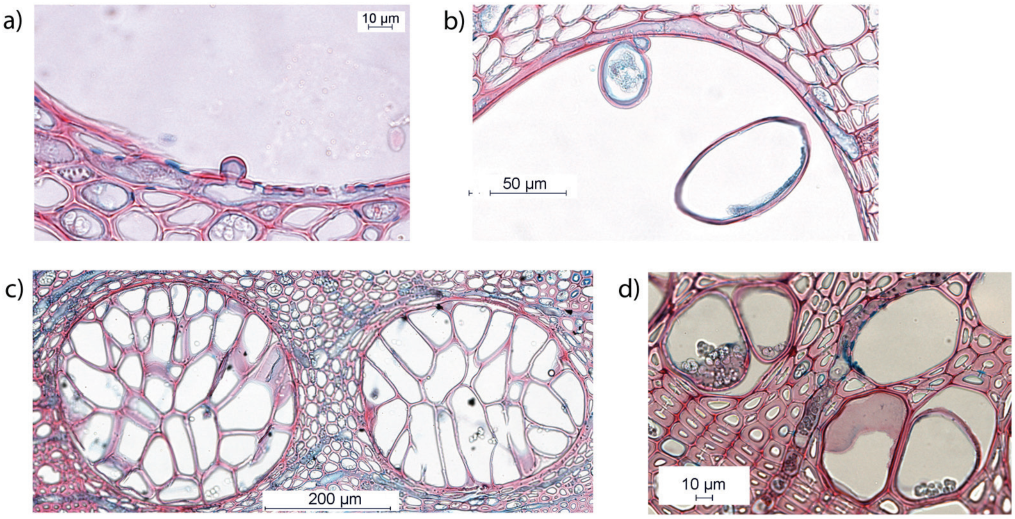

Fig. S4 Anatomical characteristics of the current-year ring once the ring was formed in *Quercus petraea*, *Fagus sylvatica* and *Picea abies* for each study year (2015–2017). BAI: basal area increment; CA: conductive area; CA:BAI: ratio between CA and BAI; D_H: Hydraulic diameter; K_h: theoretical xylem conductivity; K_s: theoretical sapwood-specific xylem conductivity. Boxplots show the median (thick horizontal line) and the first and third quartiles, with the whiskers extending from each quartile up to the highest and lowest values, respectively, or at most 1.5 of the distance between the first and third quartiles. Values beyond those limits are plotted as individual outliers. Grey letters indicate differences among species within each year, green letters differences among years in *Quercus petraea* and blue letters differences among years in *Fagus sylvatica*. The significance of inter-specific and inter-year differences was tested using generalized linear models, with differences being considered significant if $p \leq 0.05$.

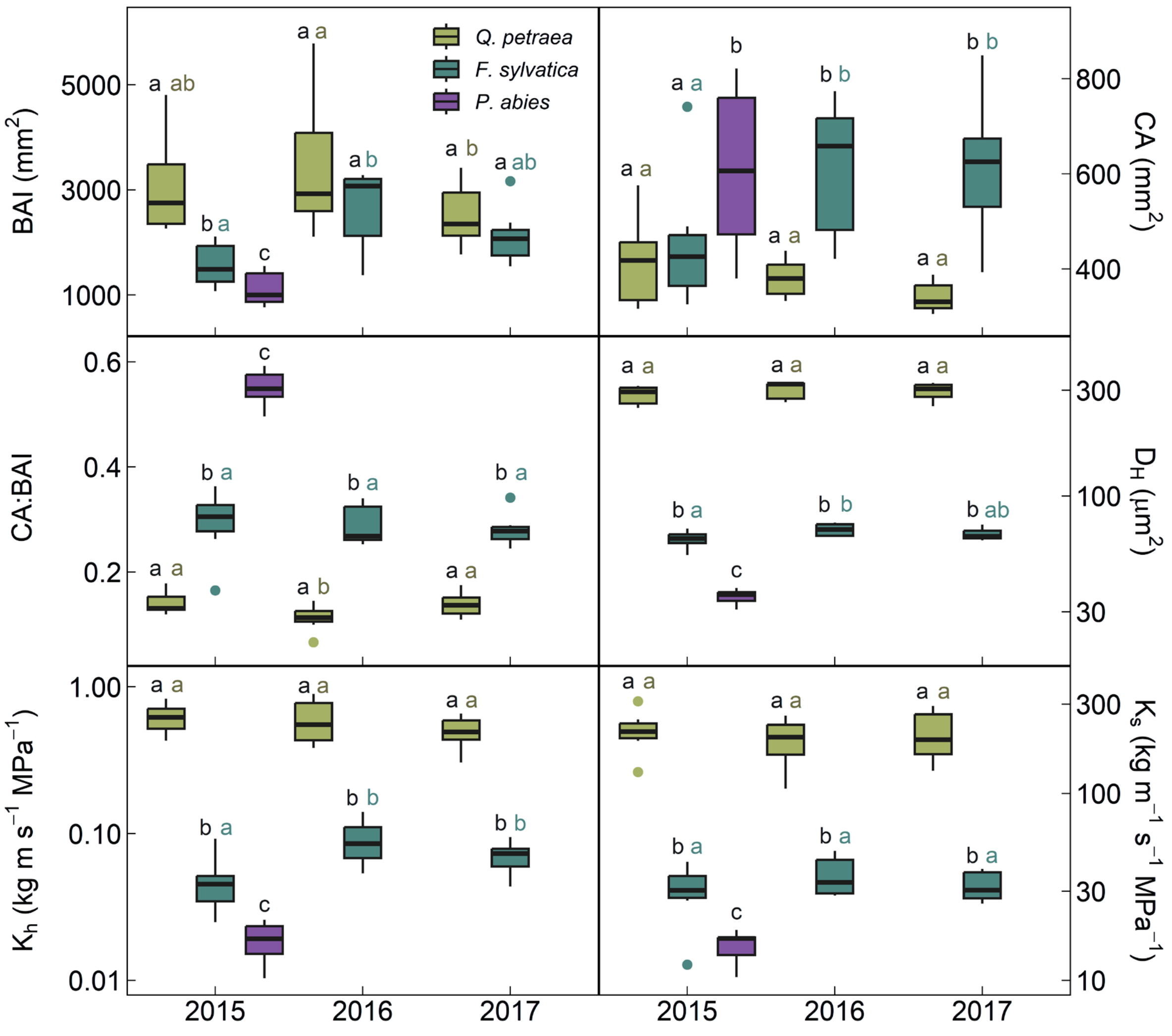

Fig. S5 Changes in mean conduit diameter, vessel composition and coefficient of variation of conduit area (CV) along the 2015 growing season in *Quercus petraea*, *Fagus sylvatica* and *Picea abies*. Vessel composition was calculated as (mean conduit area)/(number of conduits) and CV as (standard deviation of vessel area)/(mean vessel area). Points represent individual trees, with one colour per tree, and LOESS curves were fitted for each variable. Note the different y-axis scales for each species. DOY, day of the year.

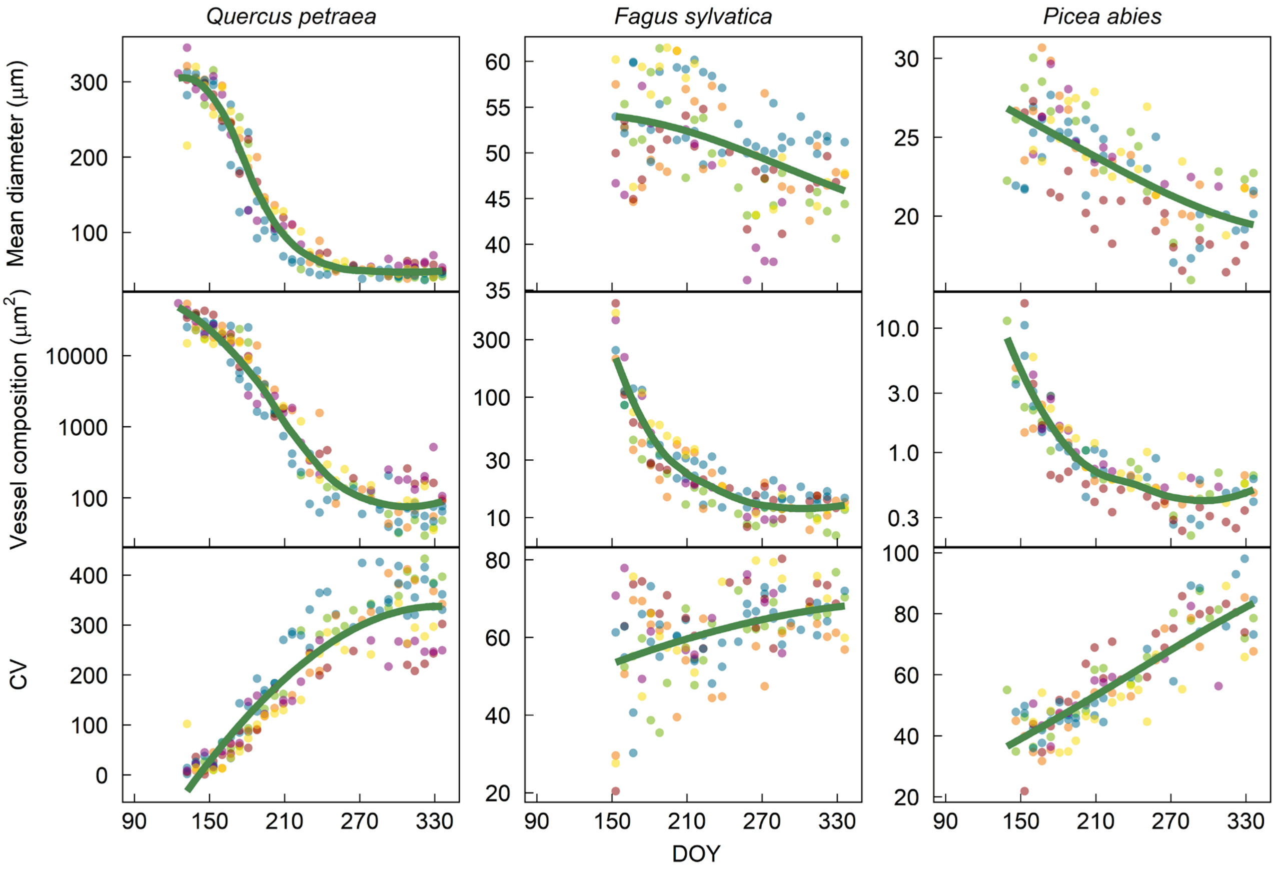

Fig. S6 Daily gain in basal area increment (ΔBAI), conductive area (ΔCA) and theoretical xylem conductivity (ΔK_h) along each study year (2015–2017) in *Quercus petraea*, *Fagus sylvatica* and *Picea abies*. Lines were smoothed by calculating the 5-day moving average. DOY, day of the year.

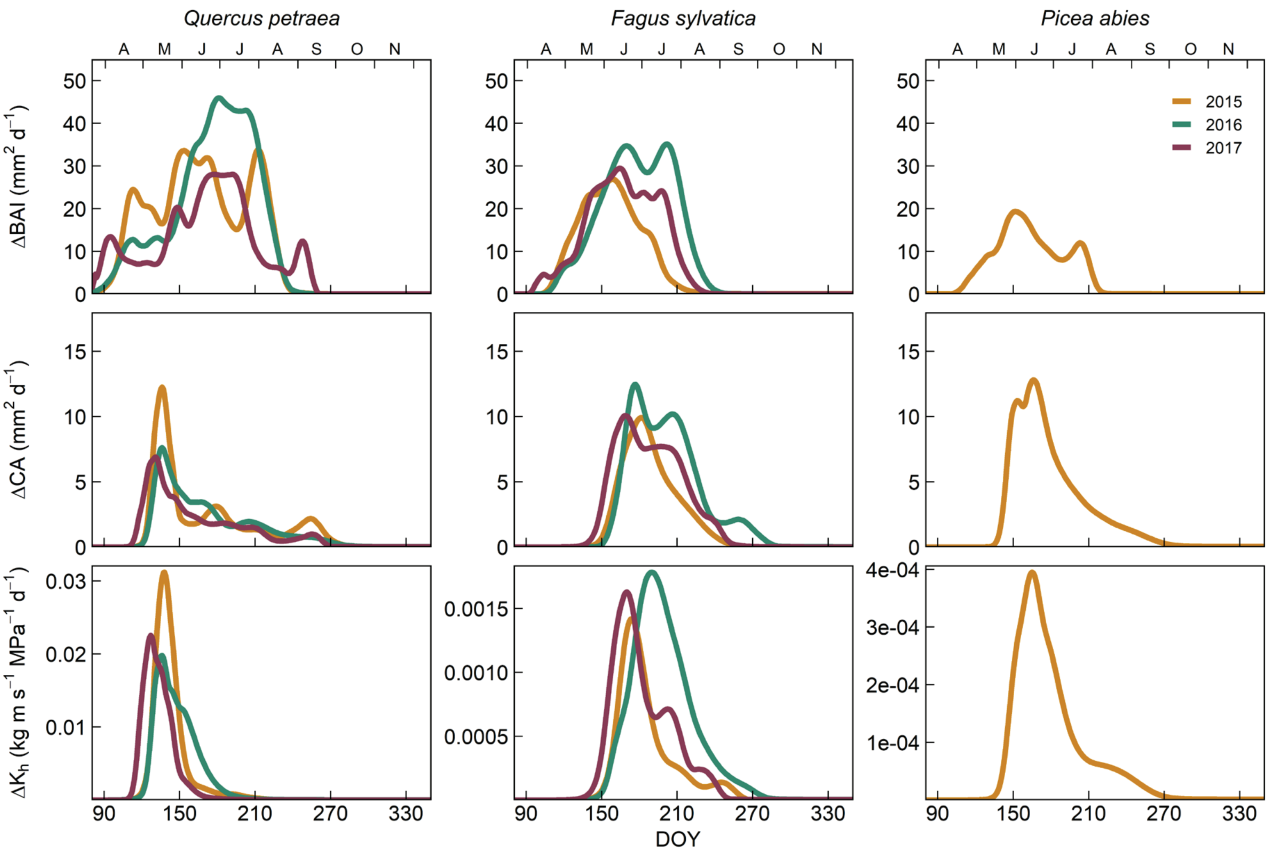

Fig. S7 Lag in days between the gain in basal area increment (BAI) and theoretical xylem conductivity (K_h) per species and study year. Point series were smoothed by calculating the 1-day moving average. Shades represent the standard error of the mean.

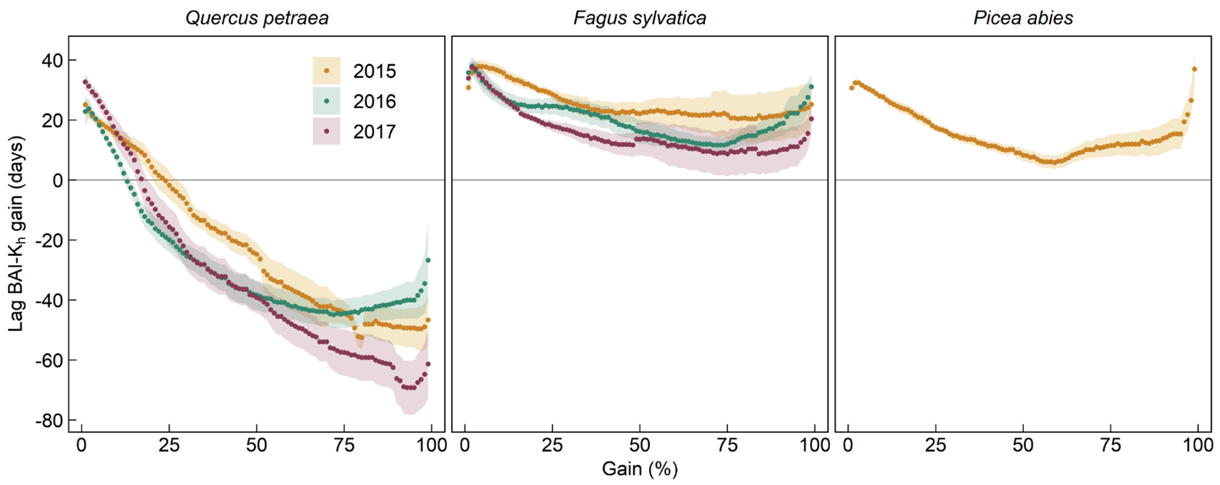

Fig. S8 Relationship between the gain in basal area (BAI) and theoretical xylem conductivity (K_h) per species and study year. Darker, straight lines represent linear mixed models of % Kh as a function of % BAI for *Quercus petraea* (0.59 + 0.44·%BAI), *Fagus sylvatica* (-0.59 + 1.52·%BAI) and *Picea abies* (-0.45 + 1.41·%BAI), with tree as a random factor on the intercept and the slope. R^2 presented correspond to the marginal R^2 of those models. The clearer, non-linear line in *Q. petraea* represents the non-linear mixed model of % Kh as a function of % BAI (0.99 - (0.99 + 8.45) $e^{-12.50 \cdot \%BAI}$). The R^2 of the fixed effects of this model (lighter font) was estimated as 1- (sum of squares of residuals/ total sum of squares).

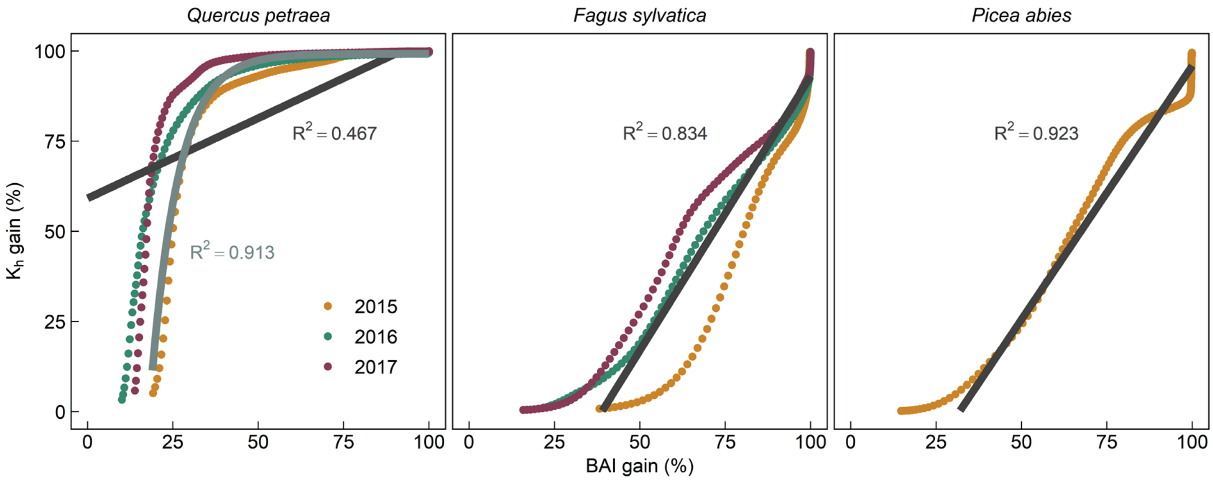

Fig. S9 Mean changes in basal area increment (BAI), conductive area (CA) and theoretical xylem conductivity (K_h) in absolute values and as a percentage of the species-specific maximum along each study year in *Quercus petraea*, *Fagus sylvatica* and *Picea abies*. Lines correspond to the average of the tree-level Shape Constrained Additive Models (SCAMs). DOY, day of the year.

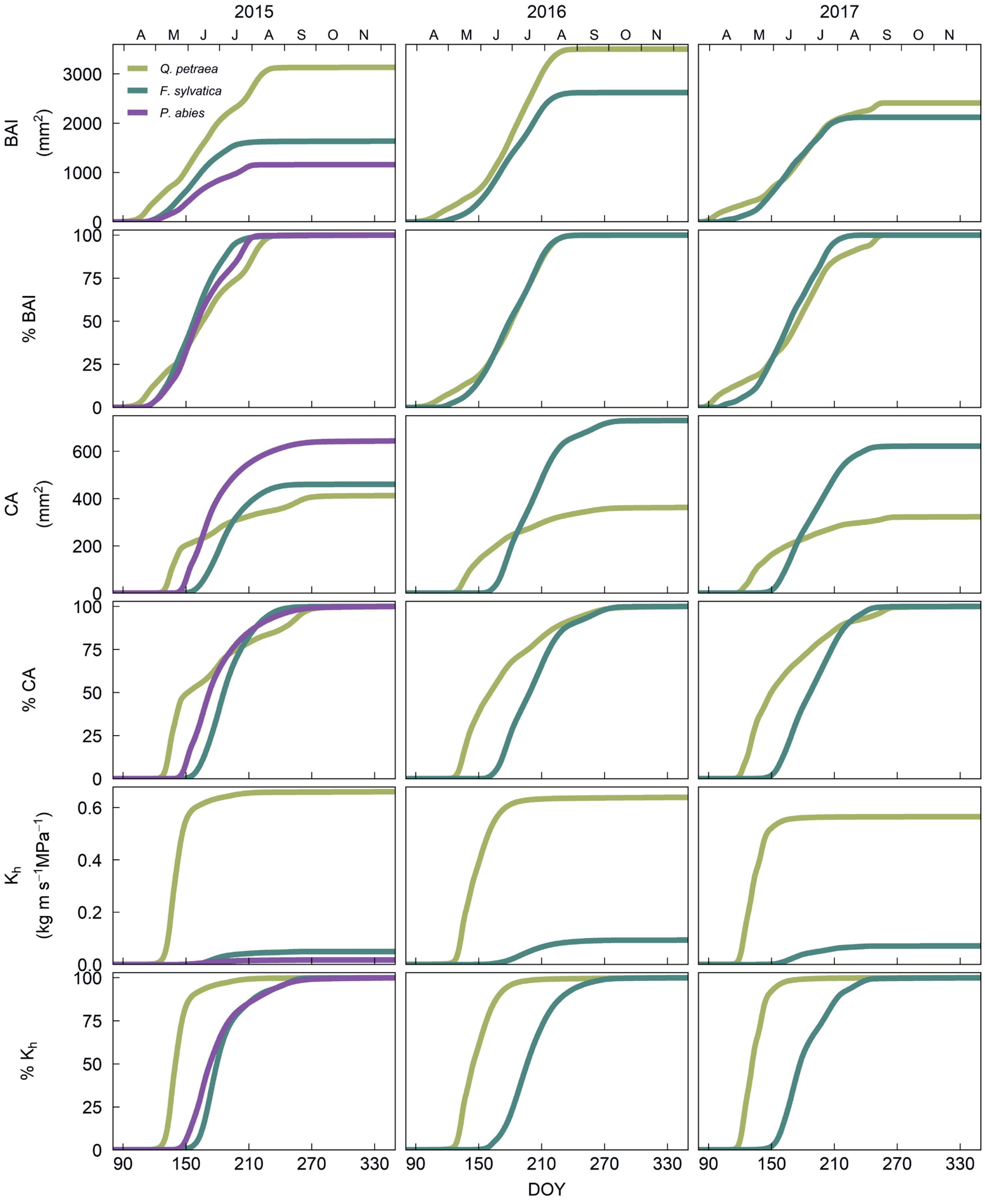

Fig. S10 Percentage of *Quercus petraea* (above) and *Fagus sylvatica* (below) trees that presented vessels with tyloses on each sampling date. DOY, day of the year.

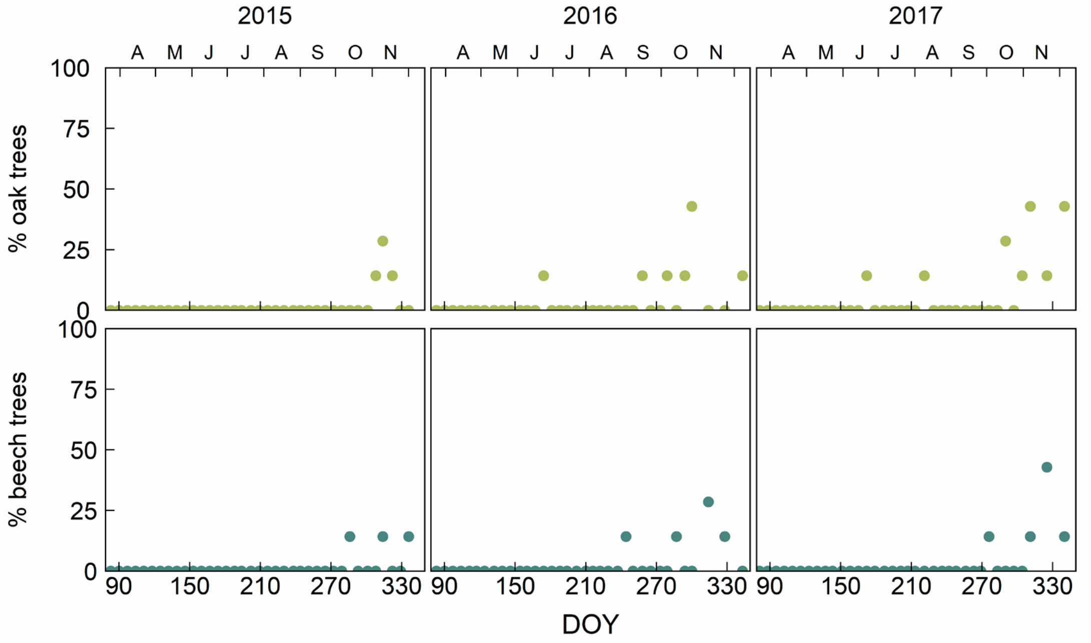

Fig. S11 Total vs functional conductive area (CA), theoretical xylem conductivity (K_h) and specific hydraulic conductivity (K_s) in *Quercus petraea* (above) and *Fagus sylvatica* (below) trees for each study year. Boxplots show the median (thick horizontal line) and the first and third quartiles, with the whiskers extending from each quartile up to the highest and lowest values, respectively, or at most 1.5 of the inter-quartile distance. Values beyond those limits are plotted as individual outliers. No significant differences were detected using generalized lineal models.

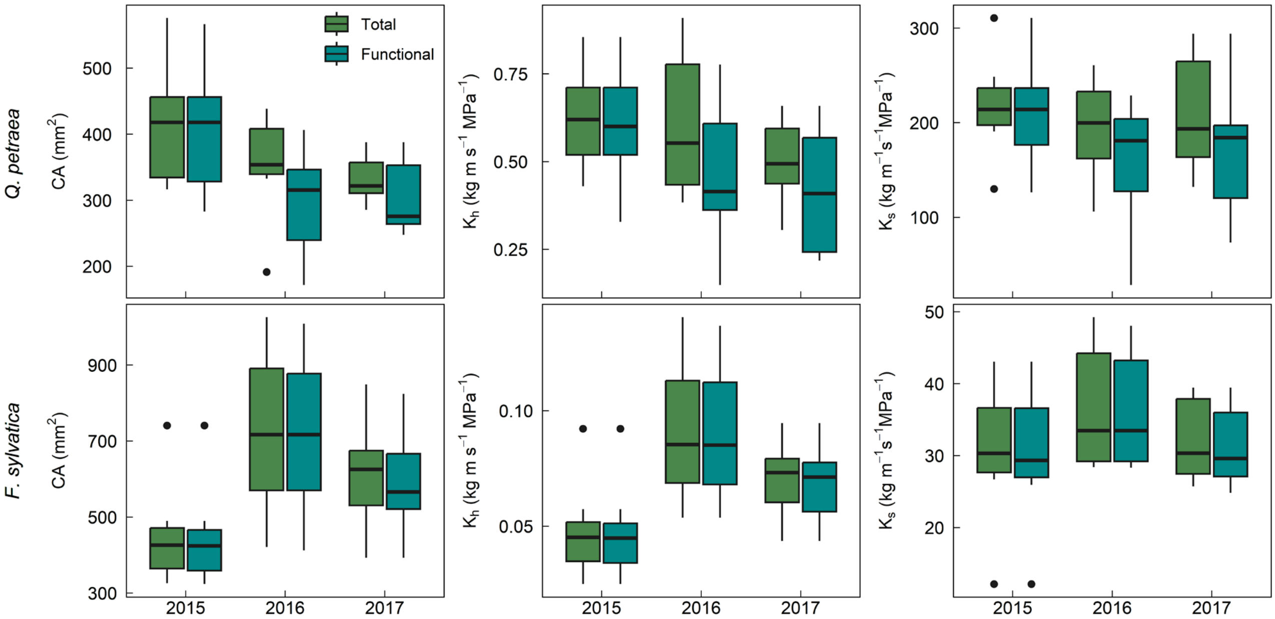

Fig. S12 Vessel diameter of vessels with and without tyloses in (a) oak's earlywood and (b) beech. Boxplots show the median (thick horizontal line) and the first and third quartiles, with the whiskers extending from each quartile up to the highest and lowest values, respectively, or at most 1.5 of the distance between the first and third quartiles. Values beyond those limits are plotted as individual outliers. Asterisks indicate significant differences ($p \leq 0.05$) per year, tested using generalized linear mixed models.

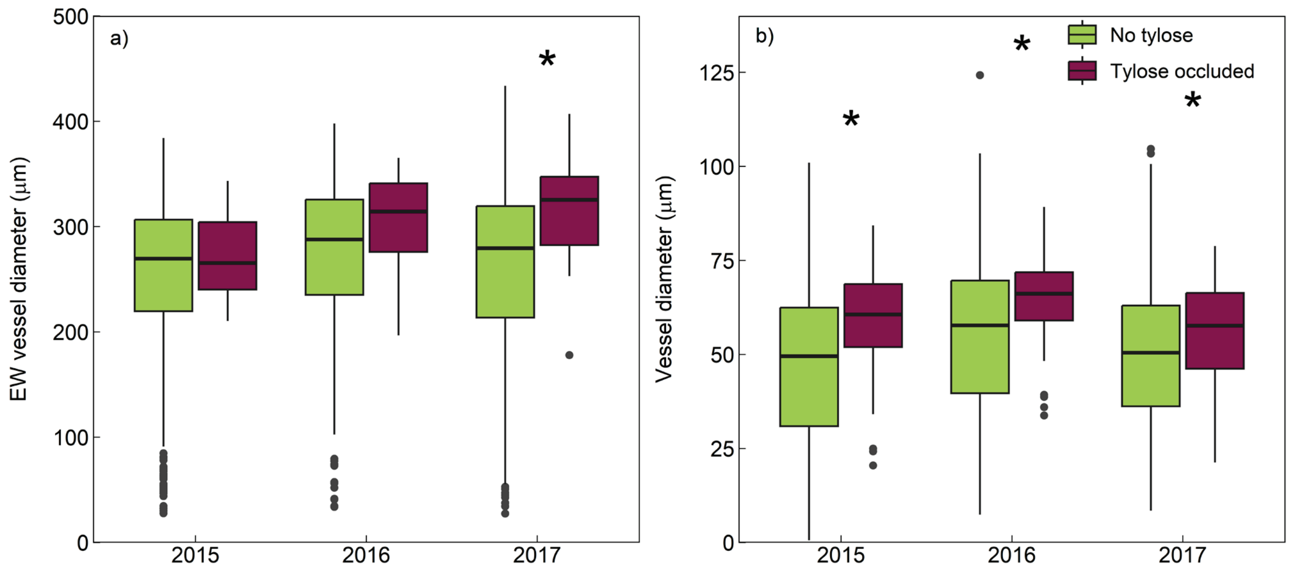

Table S1 Diameter (mean ± standard deviation, cm) of the sampled trees per species and study year.

	**2015**	**2016**	**2017**
Quercus petraea	25.8 ± 2.0	25.4 ± 3.6	25.1 ± 1.6
Fagus sylvatica	24.4 ± 2.6	27.1 ± 3.2	24.4 ± 2.1
Picea abies	28.6 ± 5.0	—	—

Table S2 Contribution (mean ± standard deviation) of earlywood vessels, latewood vessels and tracheids to total ring area, ring conductive area and ring specific hydraulic conductivity (K_s) in *Quercus petraea*. Data were obtained from two samples with fully mature xylem (one from October 2015 and another from October 2016).

**Conduit type**	**Ring area (%)**	**Conductive area (%)**	**K_s (%)**
**Earlywood vessels**	6.2 ± 2.4	40.0 ± 1.6	92.2 ± 9.4
**Latewood vessels**	4.8 ± 3.8	27.7 ± 12.1	7.6 ± 9.5
**Tracheids**	4.7 ± 0.5	32.3 ± 10.5	0.3 ± 0.1

Table S3 Reduction (in %) in functional conductive area (CA), theoretical xylem conductivity (K_h) and specific hydraulic conductivity (K_s) per species and study year as a result of vessel dysfunction as indicated by the presence of tyloses. Letters indicate differences between years within each species, whereas bold font marks differences between species per year.

	**All trees**			**Trees with tylosis**		
	**CA**	**K_h**	**K_s**	**CA**	**K_h**	**K_s**
			Quercus petraea			
**2015**	2.2 ± 5.2 a	3.8 ± 8.8 a	3.5 ± 8.2 a	7.8 ± 8.7 **a**	13.4 ± 14.4 **a**	12.4 ± 13.6 **a**
**2016**	15.6 ± 18.8 **b**	21.7 ± 26.9 **b**	22.2 ± 26.9 **b**	27.3 ± 16.7 **a**	38.0 ± 24.9 **a**	38.9 ± 24.2 **a**
**2017**	8.5 ± 8.2 ab	20.5 ± 20.2 **b**	20.7 ± 21.0 **b**	11.8 ± 7.2 **a**	28.6 ± 17.9 **a**	29.0 ± 19.0 **a**
			Fagus sylvatica			
**2015**	0.9 ± 1.2 a	1.0 ± 1.4 a	1.2 ± 1.5 a	1.6 ± 1.2 **a**	1.8 ± 1.4 **a**	2.1 ± 1.3 **a**
**2016**	0.9 ± 1.6 **a**	0.9 ± 1.5 **a**	1.0 ± 1.7 **a**	2.2 ± 2.0 **a**	2.0 ± 1.7 **a**	2.3 ± 2.0 **a**
**2017**	2.7 ± 3.3 a	2.8 ± 3.2 **a**	2.6 ± 3.0 **a**	3.7 ± 3.3 **a**	3.9 ± 3.2 **a**	3.7 ± 3.0 **a**
